# Evolutionary engineering of *E. coli* MG1655 for tolerance against isoprenol

**DOI:** 10.1186/s13068-020-01825-6

**Published:** 2020-11-09

**Authors:** Heiko Babel, Jens O. Krömer

**Affiliations:** 1 Systems Biotechnology Group, Department of Solar Materials, Helmholtz Centre for Environmental Research-UFZ, Leipziger KUBUS, Permoserstrasse 15, 04318 Leipzig, Germany; 2 Present Address: Boehringer Ingelheim Pharma GmbH & Co. KG, Biberach/Riß, Germany

**Keywords:** Adaptive laboratory evolution, *E. coli*, Isoprenol, Butanol, Tolerance, Terpenes

## Abstract

**Background:**

Isoprenol is the basis for industrial flavor and vitamin synthesis and also a promising biofuel. Biotechnological production of isoprenol with *E. coli* is currently limited by the high toxicity of the final product. Adaptive laboratory evolution (ALE) is a promising method to address complex biological problems such as toxicity.

**Results:**

Here we applied this method successfully to evolve *E. coli* towards higher tolerance against isoprenol, increasing growth at the half-maximal inhibitory concentration by 47%. Whole-genome re-sequencing of strains isolated from three replicate evolutions at seven time-points identified four major target genes for isoprenol tolerance: *fabF, marC, yghB,* and *rob*. We could show that knock-out of *marC* and expression of mutated Rob H(48) → *frameshift* increased tolerance against isoprenol and butanol. RNA-sequencing showed that the deletion identified upstream of *yghB* correlated with a strong overexpression of the gene. The knock-out of *yghB* demonstrated that it was essential for isoprenol tolerance. The mutated Rob protein and *yghB* deletion also lead to increased vanillin tolerance.

**Conclusion:**

Through ALE, novel targets for strain optimization in isoprenol production and also the production of other fuels, such as butanol, could be obtained. Their effectiveness could be shown through re-engineering. This paves the way for further optimization of *E. coli* for biofuel production.

## Background

3-Methyl-3-buten-1-ol, or isoprenol, is a hemiterpene belonging to the class of naturally occurring terpenoid compounds [1]. It is the basis for the chemical synthesis of flavor compounds, such as menthol, citral, vitamin A, E, and several carotenoids [2]. It has also been discussed for several other applications such as biofuel [3], as an anti-knocking additive in gasoline [4], and as a lead nutraceutical for longevity [5].

The biotechnological production of terpenoid compounds in microorganisms relies on the natural precursor isopentenyl diphosphate (IPP) from which isoprenol can be obtained by simple dephosphorylation. So far, strain engineering has focused on increasing the intracellular concentration of IPP. In *Escherichia coli* this has been achieved by introducing an additional metabolic pathway that produces IPP, the DXP pathway, resulting in a product titer of 61 mg/L [6]. However, the intermediate IPP has been identified as a major obstacle in those processes due to its high toxicity [7]. Using a specially designed pathway that by-passes the intermediate IPP, a product titer of 3.7 g/L was achieved. Applying the resulting strain in a fed-batch two-phase system, the highest published titer of 10.8 g/L isoprenol could be obtained [8]. Present research projects try to develop integrated processes where isopentenol (a mixture of prenol and isoprenol) is obtained from hydrolyzed polysaccharides originating from biomass [9].

Product toxicity towards microorganisms is a key issue that most economically viable bioprocesses have to face. This is also true for the biotechnological production of terpenoids and biofuels [10, 11]. Kang et al. found that indeed also isoprenol production was limited by product toxicity. A way to alleviate this toxicity is process engineering, for instance through the introduction of in situ product extraction into an organic phase. Isoprenol toxicity could be alleviated by using such a two-phase system [8], an approach that also works for other isoprenoids [12]. But two-phase systems rely on the partitioning coefficient of the target molecule between the aqueous and the organic phase. This means that the hydrophobic parts of the cell (e.g., the membrane) will most likely still be saturated with the target product and potentially the extractant, affecting structure and function. Improving the inherent resistance of the microorganism towards the target product through metabolic engineering is hence complementary to introducing in situ extraction.

Improving tolerance through metabolic engineering is possible through overexpression of export pumps [13, 14], but the prediction of engineering targets not related to transport is difficult. An approach to overcome this problem is to use the power of evolution to come up with solutions to alleviate toxicity, this can best be done by using adaptive laboratory evolution (ALE) [10, 11, 15].

In this study, we used ALE to adapt *E. coli* MG 1655 against isoprenol. We isolated highly tolerant strains that showed up to a 47% increase in growth at 50 mM isoprenol (4.3 g/L) and could grow in the presence of 80 mM isoprenol (6.8 g/L), a concentration at which the parental strain ceased to grow. With a combination of DNA and RNA-sequencing, we identified 4 target mutations for high tolerance. To confirm the role of the mutations, we re-engineered those into the parental strain and quantified the effects on isoprenol tolerance.

## Results

### Experimental evolution results in increased isoprenol tolerance

**Fig. 1:**
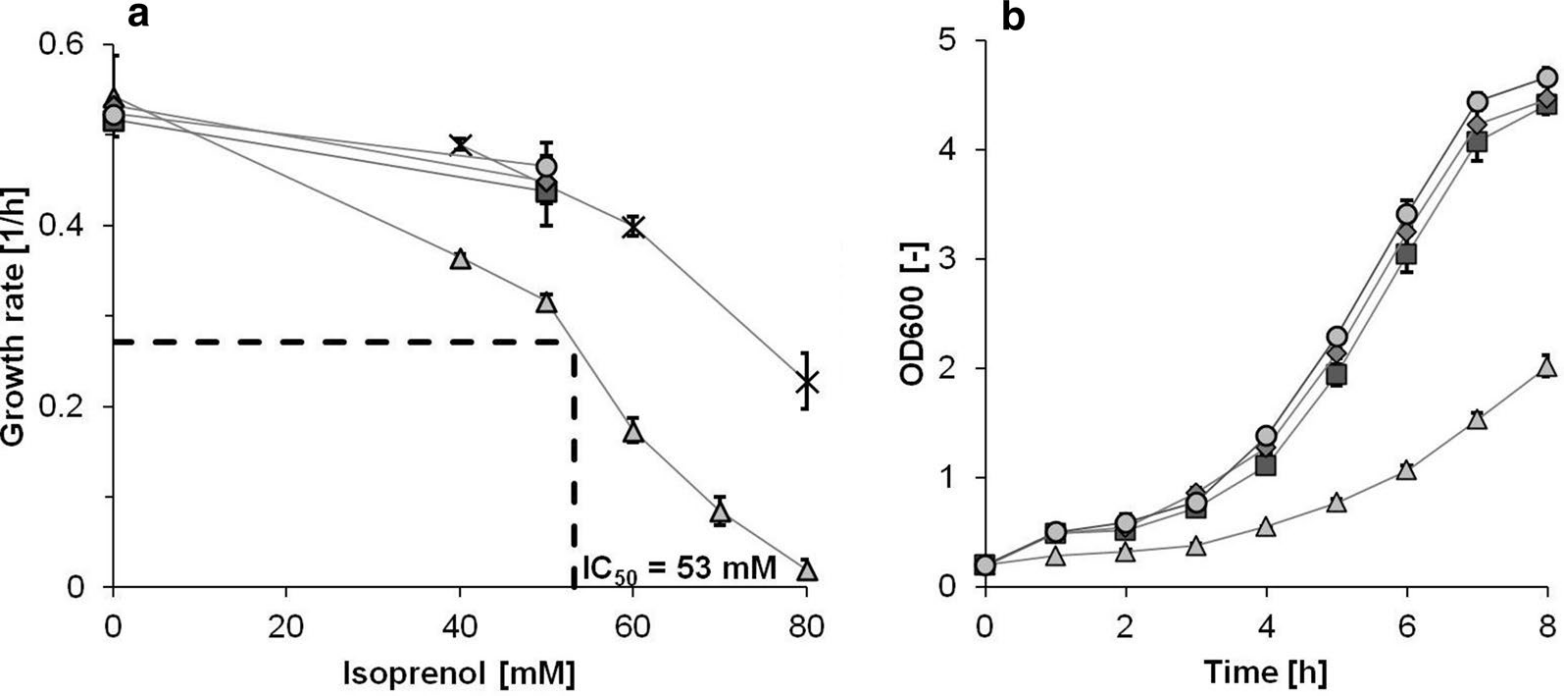
Increased tolerance against isoprenol stress of adapted strains. **a** Growth rates of parental (triangle) and adapted strains: Isolate B 100 Gen (cross), T7A (square), T7B (diamond), T7C (circle) at different isoprenol concentrations. The dashed line shows half-maximal inhibitory concentration IC_50_. **b** Growth curve of parental (triangle) and adapted strains: T7A (square), T7B (diamond), T7C (circle) in the presence of 50 mM isoprenol. Error bars represent the standard deviation of the mean of three biological replicates

To design the adaptive evolution experiment with the appropriate isoprenol stress, we first quantified its toxic effect on *E. coli* K12 MG1655 growth. We experimentally determined the half-maximal inhibitory concentration IC_50_ to be 53 mM (Fig. 1a). The growth rate decreased with increasing isoprenol concentration and there was no growth at the highest tested concentration of 80 mM.

We evolved *E. coli* by serial passaging of three independent populations for approximately 200 generations on isoprenol-spiked M9 minimal medium supplemented with 5 g/L glucose. The initial isoprenol concentration was set to 60 mM (5.2 g/L) corresponding to approximately 70% growth reduction in all three replicates. This meant that cells could grow for 24 h without reaching the stationary phase at the chosen seed-densities. During the course of the evolution, samples were taken and conserved with 50% v/v glycerol at − 80 °C. The isoprenol concentration was gradually increased to approximating a constant growth rate and reaching 80 mM after approximately 100 generations (Fig. 1a "Isolate B 100 gen"). At this time-point we already obtained tolerant strains, able to grow at isoprenol concentrations were no growth in the parental strain was observed (Fig. 1a). However, an attempt to further raise the isoprenol concentration to 90 mM after 140 generations resulted in the extinction of the cultures. The evolution was restarted from the prior cryo-stocks and from then onwards 80 mM isoprenol was used. Finally, at seven time-points during the evolution strains were isolated by selecting the five largest colonies on isoprenol-spiked LB-agar plates. The isoprenol amounts depended on the respective concentration in the evolution experiments at the time the samples were taken and were 64, 72, and 80 mM, respectively (Additional file 1: Table S1). Of those five largest colonies the fastest growing strain was determined in a falcon tube experiment with isoprenol (60 mM) (Additional file 1: Table S2) and used for genome re-sequencing.

An exact determination of the fitness benefit was performed for three final isolates of the biological replicates (Fig. 1b). At the half-maximal toxic concentration of 50 mM isoprenol, we observed an increase of up to 47% in growth rate compared to the initial MG1655. The isolated strains did not differ in their growth behavior in medium without isoprenol (Fig. 1a). Contrary to the initial strain, the final isolates could grow on LB-agar plates supplemented with 80 mM isoprenol. In summary, we successfully obtained *E. coli* strains that showed an increased tolerance against isoprenol using ALE.

### Identification of mutations by whole-genome re-sequencing

To identify the genetic basis for the increased tolerance of the adapted strain and to uncover the temporal occurrence of target mutations, genome re-sequencing was applied to the three parallel evolution experiments. There are two important points to be raised before looking at the actual results. On the one hand, the commercial isoprenol used for the evolution had 3% impurities. We found that formaldehyde was present as a major impurity, which most likely caused the mutations in the formaldehyde repressor *frmR*. We tested the final isolates against further purified isoprenol to ensure the observed phenotypes were also stable without formaldehyde. We could not observe differences in their fitness patterns. In addition, the re-sequencing revealed two strains from two parallel evolution cultures at a single time-point (culture A and C at T6) that carry the same set of four mutations. Around this time-point, all three clones isolated from the three replicates showed a great deal of exact mutational convergence, whereas most mutations previously seen in these replicates, besides *yghB*, were no longer detected. This comes after the evolution cultures had to be restarted from cryo-stocks due to extinction (see above). While this could be coincidental, it could also point to cross-contamination from another evolution. In this context, it is important to emphasize that we only sequenced a single clone per time-point and do not know the genetic heterogeneity of the cultures, but colony size on isoprenol-spiked agar plates varied (data not shown). We analyzed this computationally and were able to show that a simple model can produce our observed mutations without the assumption of cross-contamination. Adding the assumption of 5% cross-contamination modestly increases the log likelihood from − 6.1 in the model without contamination to − 2.8 in a model with contamination after T5 (which is the highest likelihood of different contamination scenarios). However, a likelihood ratio test reveals, that the differences are not statistically significant (Chi^2^-test with df = 10, p-value = 0.78). On the basis of the available data, we cannot conclude whether a cross-contamination occurred (see Additional file 1: Additional text and Tables S3–S5).

The parental MG1655, the final isolates, and isolates from the intermittent time-points of the evolution course were sequenced. All mutations identified in the re-sequencing of the isolated *E. coli* strains are listed below (Table 1) with their time-point of occurrence and their respective frequency in the whole sample of sequenced strains. In an analysis of the GO terms linked to the mutated genes, we found that target genes are associated with the membrane and fatty acid or phospholipid biosynthesis. But there were also genes acting as transcriptional regulators (Additional file 1: Table S6).

**Table 1:** Mutations that occurred in the strains isolated from the evolution experiment

Gene	Genomic coordinate	Nucleotide change	Effect of nucleotide change	Frequency (%)	Time	Description
–	257,908	G → A		5	T5	IS1 non-coding
*frmR* (1)	379,625	A → C	V(86) → G	5	T7	Formaldehyde repressor
*frmR* (2)	379,821	− 1:C	Q(21) → frameshift	5	T7	
*gltA*	754,123	C → T	E(116) → K	10	T4	Citrate synthase
*plsX* (1)	1,148,440	− 1:A	Q(274)KS → QRA STOP	10	T3	Fatty acid/phospholipid synthesis protein
*plsX* (2)	1,148,491	G → T	G(291) → C	10	T4	
*fabF* (1)	1,152,159	T → G	F(74) → C	48	T3	Component of 3-oxoacyl-ACP synthase II
*fabF* (2)	1,152,159	C → T	wt	5	T4	
*marC* (1)	1,618,245	A → T	stop → frameshift	10	T4	Inner membrane protein
*marC* (2)	1,618,498	− 7:ATCGCTA	I(135) → stop	10	T2	
*marC* (3)	1,618,805	− 1:T	M(35) → stop	48	T3	
*yffS*	2,564,930	G → T	A → A (silent)	5	T3	CPZ-55 prophage; uncharacterized protein
*yfgO*	2,615,421	G → A	A(154) → V	5	T2	Function unknown, predicted membrane permease
*iscR*	2,661,812	T → A	H(107) → L	10	T5	Iron–sulfur cluster regulator
*srmB*	2,713,364	G → A	D(157) → N	10	T2	SrmB is a DEAD-box protein with RNA helicase activity that facilitates an early step in the assembly of the 50S subunit of the ribosome
*P*_*yghB*_	3,153,480	− 15		33	T4	Required, with *yqjA*, for membrane integrity
*trkH*	4,033,611	G → A	G(156) → D	10	T5	TrkH is a potassium ion transporter
*rraA* (1)	4,119,044	A → T	V(96) → E	10	T4	RraA inhibits ribonuclease E activity by binding to and masking the C-terminal RNA binding domain of RNase E
*rraA* (2)	4,119,138	C → T	G(67) → S	5	T4	
*plsB*	4,255,502	T → C	Q(322) → R	5	T2	Membrane-bound glycerol-3-phosphate acyltransferase catalyzes the first committed step in phospholipid biosynthesis
*Rob* (1)	4,634,494	C → A	G(273) → stop	5	T5	Transcriptional regulator implied in solvent tolerance
*Rob* (2)	4,635,002	+ 1:T	Y(103) → stop	10	T6	
*Rob* (3)	4,635,168	+ 1:G	H(48) → frameshift	19	T6	
*creC*	4,637,267	T → G	L (191) → W	5	T5	Carbon source responsive sensor kinase

**Fig. 2:**
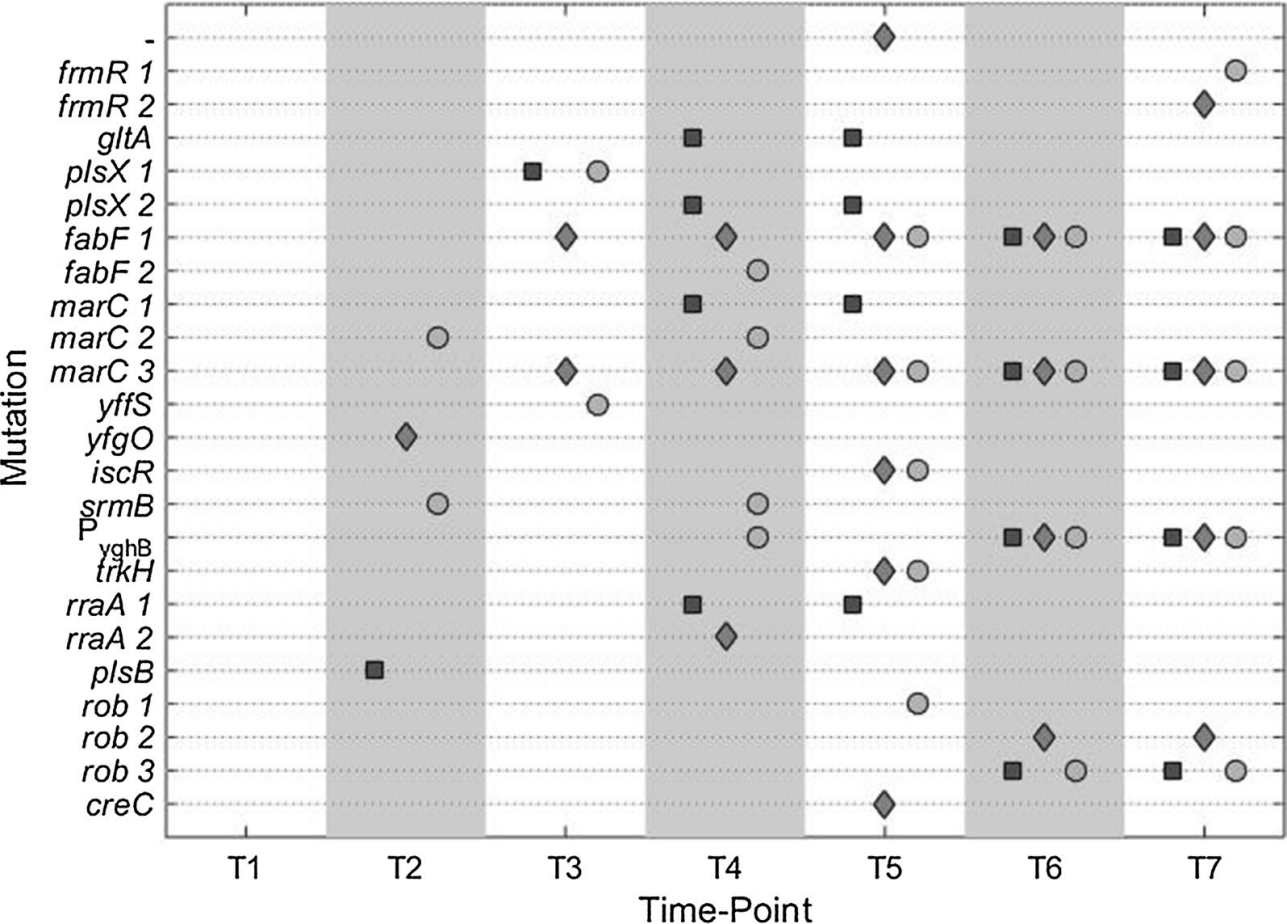
Occurrence of mutations in all isolated strains. Strains were isolated after approximately 32, 62, 108, 126, 149, 177, and 226 generations under isoprenol stress. Culture A (square), Culture B (diamond), Culture C (circle). For detailed information on mutations refer to Table 1

Protein functions are taken from ecocyc.org. For genes with alternative mutations, the numbering behind the gene name refers to the mutation name provided in Fig. 2

### Occurrence and persistence of mutations in the evolution experiment

To visualize the evolution of the three cultures and identify persistent mutations, we plotted the mutations in the different cultures in their temporal order in Fig. 2. In the isolates at the first time-point approximately after 32 generations, we could not identify any mutations. In the second isolates, the first mutations occur, but none of those persist. In the isolates after 108 generations the FabF F(74) → C and MarC M(35) → stop mutation appear and in culture B persist until the end of the evolution experiment. At the next time-point T4, a mutation upstream of *yghB* appears that is also present in the final isolates. Finally, the first mutation in the *rob* gene appears at T5, in culture C. After T6 all isolated strains carry the *fabF* mutation, the *marC* mutation, and the *yghB* promoter mutation. Also, all strains isolated at T6 and T7 have one of two mutations of the *rob* gene. In two of three strains isolated at the final time-point, there are also two distinct mutations in the *frmR* gene. All other mutations that occurred in the intermediate isolates were not found in the final isolates, i.e., they did not persist. Interestingly in culture A, we isolated apparently the same genotype at T4 and T5 twice carrying mutations in the genes *gltA*, *plsX*, *marC,* and *rraA*. Of those target genes, *marC* is also found in the final genotype, also distinct *plsX* and *rraA* mutation can be identified in other isolates at other time-points making them additional targets for the tolerance phenotype.

### Transcriptional response of adapted strains

To understand how the genetic adaptations influence the cell's phenotype, we performed an RNA-Seq experiment with the parental strain and strains isolated at T7. All strains were put under isoprenol stress (50 mM) and subsequently, mRNA levels were quantified. We were primarily interested in the transcriptome changes that are consistent among all three mutant strains since the final isolates share three exact mutations and one target gene.

The top ten up- and down-regulated transcripts compared to the parental strain are listed below (Table 2). Similar to the target genes of the mutations, a gene ontology analysis revealed that highly differentially regulated transcripts were associated with the cell membrane and integral membrane components (Additional file 1: Table S7). Mutations in three genes were observed, that could directly affect the regulation of gene expression: FrmR is a transcriptional repressor, the mutation at the upstream of *yghB* could influence its expression and finally, Rob is a global transcriptional regulator. For the *yghB* gene, we found in all samples a strong up-regulation in the isolated strains compared to the parental strain. For both strains with a mutation in the *frmR* gene, we observed an up-regulation of the *frmRAB* operon compared to the parental strain (data not shown). Most of the strongly differentially regulated transcripts were not directly linked to genetic mutations found in the isolates. For instance, a very strong down-regulation was also observed for *hslU* (Table 2). HslU is part of the protease system responsible for the degradation of the Arc repressor, usually induced by heat-shock.

**Table 2:** Mean transcriptional response of adapted strains T7A-C against Isoprenol stress compared to the parental strain MG1655

Transcript name	Cuffdiff	DESeq	edgeR	Description
*ompF*	3.66	5.52*	5.76*	Outer membrane protein F (Porin)
*alaE*	5.20*	3.60	5.24	Ala-Ala exporter
*yghB*	3.87*	3.66	3.84*	Membrane protein
*ilvX*	2.18	3.07*	3.77*	Unknown
*ilvG, ilvM*	2.41*	2.26*	2.72*	Val and Ile synthesis
*ldrA*	0.00	1.19	4.94*	Peptide toxin
*glxK*	1.93*	1.11	1.62	Glycerate kinase II
*yahO*	1.69*	1.35	1.55*	Tolerance against X and UV radiation
*yhaH*	1.59*	1.30	1.55*	Inner membrane protein
*yodC*	1.68*	0.78	1.40	Fimbrial tip-adhesin
*ynfQ*	− 3.06*	− 1.86	− 3.25*	Small protein cold shock-induced
*citC*	− 2.77	− 2.72	− 2.97*	Citrate lyase synthetase
*glgS*	− 3.20*	− 2.86	− 3.09*	Regulates motility and biofilm
*flu*	− 3.29*	− 3.07	− 3.25*	Small RNA, membrane protein, aggregation
*cspI*	− 3.64*	− 2.60	− 3.51*	Cold-shock protein
*isrC*	− 3.59*	− 3.17*	− 3.49*	Small RNA
*menA*	NaN	− 6.19*	− 11.32*	Membrane protein
*hslU*	NaN	− 8.74	− 14.37*	ATPase component of HslVU protease
*sapB*	− 13.57	− 7.54	− 15.13*	Membrane subunit of putative putrescine exporter
*rraA*	− 13.13	− 9.27*	− 14.45*	Inhibits ribonuclease activity

Log2 fold-changes were determined using cuffdiff, DESeq, and edgeR, values are mean fold-change of strain T7A, T7B, and T7C compared to the parental strain. Asterisks indicate statistically significant differential regulation (*p* < 0.05) for all three adapted strains. Protein functions are taken from ecocyc.org

### Characterization of mutations

Finally, we wanted to analyze the contribution of each highly persistent target mutation to the tolerance phenotype, except for the highly prevalent *fabF* mutation. This has already been discovered and analyzed previously in a mutagenesis study for butanol tolerance [16]. Because of the similarity of isoprenol and butanol in terms of structure and polarity, we did not test the *fabF* mutation under our conditions.

#### yghB

**Fig. 3:**
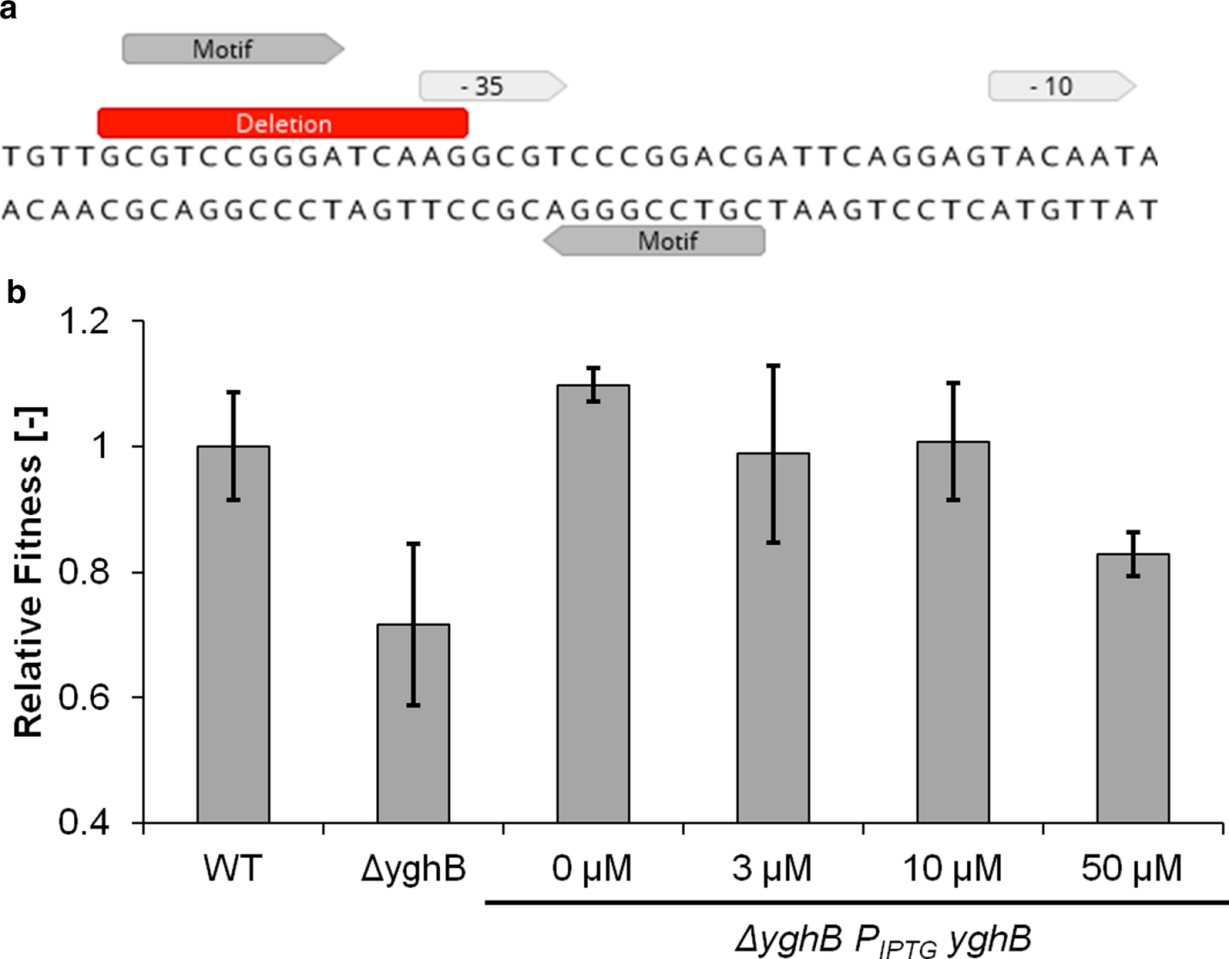
Reconstitution of *yghB* mutation. **a** Deletion upstream of *yghB* and annotation of putative regulator motif. **b** Complementation of *yghB*-knock-out mutation with a plasmid carrying *yghB* under control of an inducible promotor (IPTG) under isoprenol stress (50 mM). Relative fitness is defined as the observed growth rate divided by the growth rate of the initial MG1655 (WT) under identical conditions. Values represent the mean value of three biological replicates. Error bars indicate standard deviation

The mutation found upstream of the *yghB* gene deleted a 15-bp portion that overlaps with the − 35 region of the promoter. Interestingly there appears to be a 9-bp DNA motif that lies in the deletion region and is repeated inverted after the − 35 region (see Fig. 3a). Those repeated regions might be the binding site of an unknown transcriptional regulator or might form a secondary structure. In the case of an activator, deletion would reduce *yghB* expression in case of a repressor the opposite would be the case. In fact, the RNA-Seq data showed that *yghB* was 14-fold upregulated (Additional file 1: Figure S1) compared to the parental strain suggesting the deletion of a repressor binding site.

Knock-out of *yghB* (strain *ΔyghB*) clearly increased the sensitivity against isoprenol (Fig. 3b), while introducing a *yghB* carrying plasmid into the knock-out strain (strain Δ*yghB yghB*) complemented the effect. Due to the leakiness of the promotor on the high-copy number plasmid, induction was not even necessary.

#### rob

**Fig. 4:**
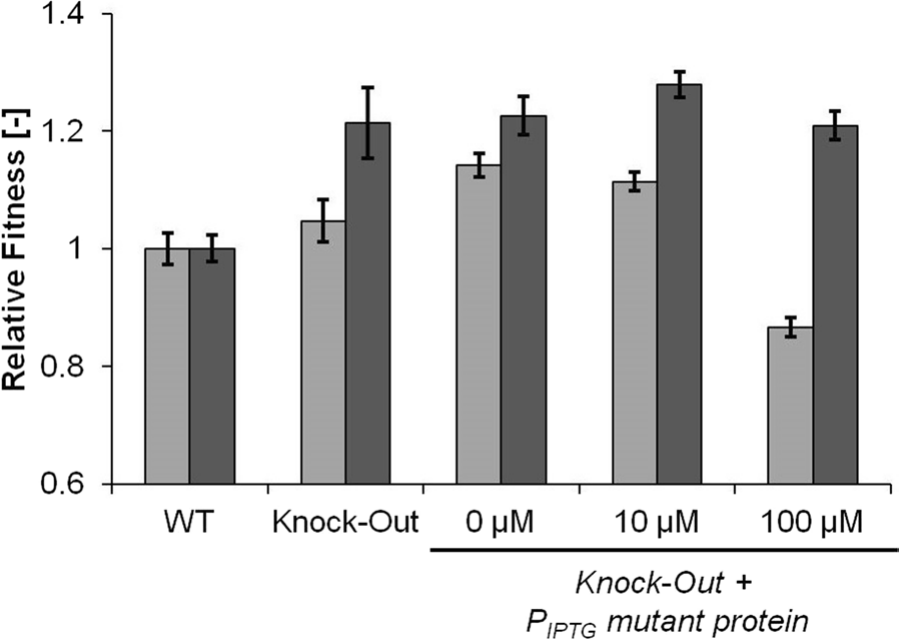
Complementation of Δ*rob* (light grey) and Δ*marC* (dark grey) with IPTG inducible rob mutant H48frameshift or marC mutant M35stop, respectively. Relative fitness is defined as the respective observed growth rate divided by the growth rate of the initial MG1655 (WT) at 50 mM isoprenol. Values represent the mean value of three biological replicates. Error bars indicate standard deviation

The Rob protein is a transcriptional activator that regulates together with SoxRS and MarA a stress response regulon [17]. In the evolution experiment, we identified three variant mutations G(273) → *stop*, Y(103) → *stop* and H(48) → *frameshift* which appeared in two final strains and in total with the highest frequency. The fact that with increasing frequency of the *rob* mutations less of the protein should remain functional led us to hypothesize that the fitness benefit originated from a loss of function. Consequently, we tested the fitness of a *rob* knock-out (strain *Δrob*) with isoprenol stress, but found no increase in fitness (Fig. 4 light gray). The mutated Rob H(48) → *fs* protein might retain some of its N-terminal DNA-binding domain and thereby still have a function as a transcription factor with a different or no sensory response since its C-terminal domain cannot have its original function due to the truncation. Plasmid-based introduction of the mutated RobH(48) → *fs* protein (strain *Δrob robH*) increased the fitness approx. 14%. At a low induction level of 10 µM isopropyl-β-d-thiogalactopyranoside (IPTG) there was still a positive fitness effect; the fitness of the strain became negative at high induction levels of 100 µM IPTG. Since the knock-out of *rob* did not have a fitness benefit and expression of the mutant protein did, it appeared that the mutated version had an altered function in the cell that benefitted isoprenol tolerance. The RNA-sequencing analysis showed that indeed only 3 out of 21 genes of the rob-regulon (*acrZ*, *yhbW,* and *inaA*) were significantly down-regulated in all mutant strains (Additional file 1: Figure S2), while other genes of the regulon remained unchanged or even slightly upregulated. AcrZ is a membrane protein associated with an efflux pump [18]. Rob-controlled expression of *inaA* was observed during dipyridyl stress [19] and *yhbW* has been predicted to be a monooxygenase [20] and was shown to be strongly upregulated during ALE experiments on vanillin but without activity against vanillin [21].

#### marC

MarC is a transmembrane protein [22] and a positive effect of gene deletion has been found in isobutanol tolerance [11]. Minty et al. found a disruption of the *marC* locus by a transposon insertion and hypothesized that this insertion would have a positive effect. Indeed a knock-out of *marC* (strain *ΔmarC*) resulted in increased isobutanol tolerance. In our experimental setup knock-out of *marC* also had a positive effect on isoprenol tolerance (Fig. 4). Since the highest frequency mutation of *marC* introduced a stop codon after the 35th methionine residue, we wondered whether the protein fragment had an additional fitness benefit. Expression of the MarC M(35) → *stop* mutant in the *marC* knock-out background (strain *ΔmarC marC35*) did not significantly change the fitness compared to the knock-out (Fig. 4) and appeared to only lead to a loss of MarC function.

### The extent of the tolerance mechanism to additional compounds

**Fig. 5:**
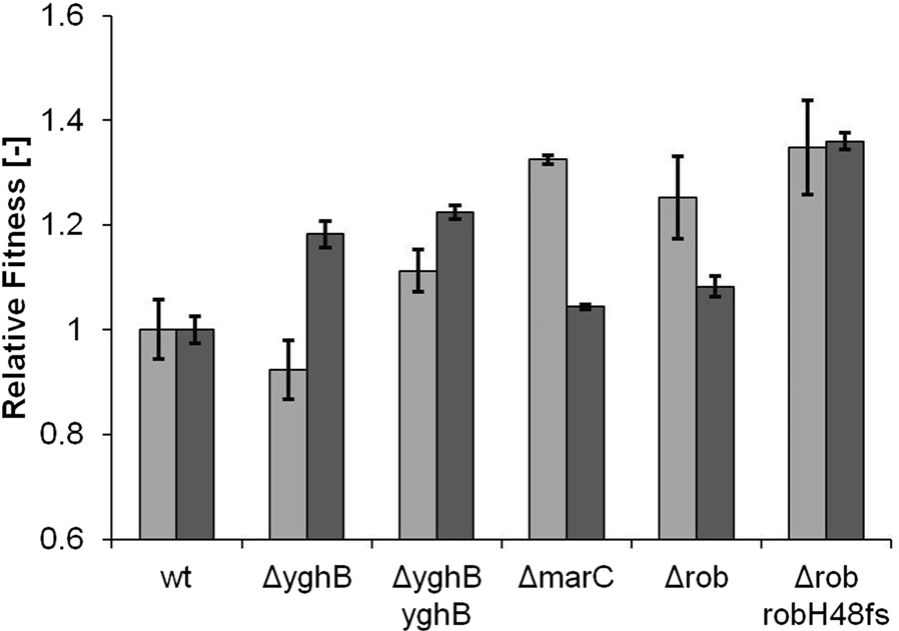
Relative fitness, as described above, against butanol (5 g/L) (light grey) and vanillin (1.5 g/L) (dark grey) of the different reconstructed strains. MG1655 growth rate was 0.25 1/h and 0.24 1/h, respectively. Values represent the mean value of three biological replicates. Error bars indicate standard deviation

In our analysis, we found that some of the target genes were identified in evolution experiments against C-4 alcohols. Therefore, we tested the novel mutations for their fitness benefit against butanol (Fig. 5). Knock-out of *yghB* resulted in a slight fitness decrease. Analogously to isobutanol tolerance a *marC* knock-out also increased tolerance against butanol. In contrast to isoprenol tolerance, knocking out the regulator *rob* already had a strong fitness benefit. Introducing the plasmid expressing the *rob* H(48) mutant only raised fitness slightly. For butanol tolerance, the mutations found in the isoprenol evolution behaved mostly similar, except for *rob* where a knock-out did not improve tolerance against isoprenol.

Finally, we wanted to test a compound with different physicochemical properties and evaluate if still the same mutations benefit tolerance. In earlier experiments, we observed no improved tolerance (data not shown) of our evolved strains towards geraniol (logP 2.5, solubility in water 0.68 g/L), another terpenoid of commercial relevance. We chose vanillin (logP 1.37, solubility in water 10 g/L) as a compound with a logP value between geraniol and isoprenol to test our target mutations (Fig. 5). We found that the *yghB* gene still played a significant role for tolerance, however, its role was the opposite compared to isoprenol and butanol tolerance since the knock-out had a positive fitness effect. Knock-outs of *marC* and *rob* did not increase fitness strongly. But a strong fitness benefit could be obtained by expression of the mutant *rob* H(48) protein, a similar effect as observed on isoprenol tolerance.

## Discussion

Using an ALE approach, we were able to identify strains that are highly tolerant against isoprenol compared to the parental *E. coli* MG1655 strain. At the IC_50_ the initial MG1655 strain was 50% inhibited, while in the isolated strains exhibited growth at 75% of the unchallenged maximum. At the same time, these strains were capable of growing at 80 mM isoprenol a concentration completely inhibiting the parental MG1655. By re-sequencing multiple strains isolated throughout the evolution, we could identify approximate time-points of the appearance of mutation and calculate their persistence. Performing the same analysis on three biological replicates further increased our ability to judge the importance of certain mutations. The combination of genome re-sequencing with RNA-sequencing made it possible to determine the regulatory effects of mutations that are in the intergenic region.

### Evolution

The temporal order of those key mutations could hint at strong epistatic effects, i.e., that fitness benefits of a late mutation such as P_*yghB*_ are much stronger in a *marC* background. In addition, the temporal order could be the consequence of increasing isoprenol concentration and thereby increased stress during evolution. If this would be the case, late mutations would exhibit a much larger fitness increase under higher isoprenol concentrations. However, it was beyond the scope of this study to test epistatic and stressor concentration-dependent fitness effects systematically. We find that at the half-maximal isoprenol concentration of 50 mM, the mutations tested in this study (P_*yghB*_, Δ*marC,* and Rob H(48) → fs) would have an additive relative fitness that overlaps with the fitness benefit of the final isolates that have the additional *fabF* mutation. This is an indication that at this isoprenol concentration epistatic interactions must be negative, otherwise, at least one mutation would not have persisted in the evolution [23, 24].

We observed no mutations at the first time-point of isolation (32 generations) although our growth data indicated already an improvement of growth on isoprenol (Additional file 1: Table S1). This means that in addition to genetic adaptation, a phenotypic or possibly epigenetic adaptation occurred.

### Contribution of single mutations to tolerance

Two of the target genes in the set of highly persistent mutations have been identified in previous studies. The MarC membrane protein had initially been assumed to play a role in multiple antibiotic resistance [22]. In an adaptive evolution against isobutanol, Minty et al. discovered an insertion element in the *marC* gene and confirmed that a knock-out of *marC* provided increased tolerance against isobutanol. Similarly, Atsumi et al. found the deletion of the complete *mar* operon to provide isobutanol tolerance [25]. We showed in our study that knock-out of *marC* also increases tolerance against isoprenol and expression of the mutated MarC M(35) → *stop* does not have a strong additional fitness effect.

A mutation in the *fabF* gene resulting in an amino acid exchange F(74) → C occurred with a high frequency in our re-sequencing dataset. The identical mutation has previously been identified in a mutagenesis study of *E. coli* for butanol stress [16]. In a subsequent study, the investigators showed that the amino acid change led to a change in fatty acid composition increasing the major unsaturated fatty acid, *cis*-vaccenic acid [26].

Following the *fabF* and the *marC* mutation, the deletion upstream of *yghB* occurred with the second-highest frequency. YghB belongs to the DedA protein family of membrane proteins and is necessary for temperature tolerance [27]. It was speculated that *yghB* and its family members are proton-dependent transporters [28]. The analysis of the deleted sequence and its context revealed that upstream of *yghB* an unknown 9 bp sequence motif is present that is repeated as an inverted repeat. Interestingly, part of the motif overlaps with the -35 region possibly obstructing binding of the sigma-factor; the motif could be recognized by a repressor protein or inhibit transcription by cruciform extrusion [29]. Knock-out of *yghB* decreases isoprenol tolerance showing its relevance for the tolerance mechanism. The knock-out could be complemented with *yghB* on an inducible plasmid. The *yghB* promoter mutation also showed significant effects on downstream genes. In fact, the RNA-seq data showed that especially *yqhC* and *yqhD* were also significantly upregulated (Additional file 1: Figure S2).

The third most abundant gene target *rob* belongs to the AraC/XylS protein family of transcription factors [30]. It has been implicated in organic solvent tolerance [31, 32] and together with MarA and SoxS it regulates a large regulon [17]. Interestingly, complementary tolerance engineering and functional genomic approaches yielded gene targets such as *soxS* and *marA* [33, 34] that share a regulon with *rob* via the marbox DNA motif [35]. Rob consists of two major domains, an N-terminal DNA-binding domain comprised two HTH motifs and a C-terminal domain that binds to its signal molecules and is able to form protein complexes. The G(273) → *stop* mutation shortened the C-terminal domain, Y(103) → *stop* truncated it completely. These mutations might lead to a signal independent activation of the Rob-regulon [17]. The most prevalent mutation, H(48) → *fs*, however, resulted in an altered protein after the first of the two HTH motifs of the N-terminal DNA-binding domain [30]. Structural data shows that both HTH motifs of Rob bind to DNA [30], the Rob H(48) → *fs* might therefore not be functional. However, we could show that a *rob* deletion mutant did not have a fitness benefit, instead a plasmid-based expression of the mutated version of *rob* lead to a significant fitness increase. *rob* has been shown to trigger the expression of the AcrAB multidrug efflux pump and confer tolerance to multiple substances [36]. However, our RNA-sequencing analysis showed that *acrAB* expression was not significantly changed in the evolved strains and that the multidrug efflux pump accessory protein gene *acrZ* was even down-regulated (Additional file 1: Figure S2). The *marR* regulator is also part of the *rob* operon (Additional file 1: Figure S2) and was slightly downregulated (FC around − 0.8). This did, however, not lead to a significant change in the expression of *marABC* (data not shown). How exactly this mutant Rob protein binds to DNA and how the altered structure results in an altered function remains unclear and needs to be addressed in future studies.

Both *rraA* and *plsX* are targets of two distinct mutations and occur in conjunction in an intermediate genotype of culture A. *rraA* is an inhibitor of RNase E and globally influences RNA stability [37]. The more prevalent mutation V(96) → E introduced an amino acid change in the highly conserved V(96) residue [38] and possibly had a deleterious effect. Such a mutation would consequently globally increase mRNA levels. Possibly isoprenol also targets the intracellular translation process which could be compensated for by increasing mRNA levels. Interestingly, *rraA* was the transcript that was downregulated the strongest (up to 2^–14^-fold) in the isolated final mutants compared to the initial strain.

The fatty acid/phospholipid synthesis protein PlsX has also been identified as a target in an evolution study against isobutanol [11]. In our study, two mutations that locate to the c-terminal α-helices were identified [39]. The C-terminal parts of *plsX* are rather conserved and important for dimerization [39], amino acid changes might be deleterious or change the substrate specificity thereby changing the lipid composition in the cell membrane.

### Tolerance against additional compounds

The mutations investigated in this study could also be used to engineer tolerance against butanol stress. Especially, *yghB* overexpression and expression of mutant *rob* lead to significant fitness increase. The fragrance compound vanillin appeared to have different toxic effects on MG1655, here surprisingly deletion of *yghB* increased the growth rate by approximately 20%. A knock-out of *marC* did not increase fitness, however, the mutant *rob* had a strong fitness effect. We found that similar gene targets play a role in tolerance against structurally different compounds such as vanillin although in a qualitatively different manner.

## Conclusions

ALE is a powerful tool to be used alongside strain development, especially for the development of chemical resistance. It has been shown previously that the toxicity of isoprenol is the current limitation for high-yield production [8]. With our experimental evolution approach, we could identify four target mutations that could be implemented in isoprenol producing *E. coli* strains in the future to alleviate the toxic effects of the product. The same mutations also showed effects in *E. coli* during challenges with butanol or vanillin, albeit to a different extent.

## Materials and methods

### Chemicals and reagents

All chemicals used in this study were at least of analytical grade and purchased from Sigma-Aldrich. Aqueous stock solutions were made using ultra-pure water (resistance > 18 MΩ). Isoprenol was also purchased from Sigma with 97% purity. BASF identified formaldehyde as an impurity. To ensure strains were not selected on formaldehyde resistance only, the final isolates were also challenged with isoprenol depleted of formaldehyde (custom chemical provided by BASF SE). No effect on resistance could be observed.

### Strains

The *E. coli* MG1655 (CGSC6300) strain [40] used in the evolution (GenBank: GCA_000005845.2) exhibited a reconstituted *gatC* gene, a functional *glrR* glycerol 3-phosphate repressor [41] and variation in the repeat REP321j. The following strains were used and constructed in this work (Tables 3 and 4):

**Table 3:** Background strains

Strain	Genotype	References
*Escherichia coli* MG1655	K-12 F^–^ λ^–^ *ilvG*^–^ *rfb-50* *rph-1*	[40]
*Escherichia coli* DH5α	F– *endA1 glnV44 thi-1 recA1 relA1 gyrA96 deoR nupG purB20 φ80dlacZΔM15 Δ(lacZYA-argF)U169, hsdR17(rK–mK*+*), λ–*	–
*Escherichia coli* BW25113	rrnB3 DElacZ4787 hsdR514 DE(*araBAD*)567 DE(*rhaBAD*)568 rph-1	[42]
Keio Δ*marC*	BW25113 *marC::kan*^*R*^	[42]
Keio Δ*rob*	BW25113 *rob::kan*^*R*^	[42]
Keio Δy*ghB*	BW25113 *yghB::kan*^*R*^	[42]

**Table 4:** Strains constructed in this work

No.	Strain genotype	Resistance	Alias
557	*E. coli* MG1655 *Ptrc*10 *yghB*	*ampR cmR*	*yghB*
564	*E. coli* MG1655 P*trc*10 empty	*ampR cmR*	Empty vector
952	*E. coli* MG1655 Δ*yghB*781::*kanR*	*kanR*	*ΔyghB*
953	*E. coli* MG1655 Δ*marC*750::*kanR*	*kanR*	*ΔmarC*
954	*E. coli* MG1655 Δ*rob*-721::*kanR*	*kanR*	*Δrob*
960	*E. coli* MG1655 Δ*marC*750::*kanR Ptrc*10 *marC*35 *kanR*	*kanR ampR cmR*	*ΔmarC marC35*
961	*E. coli* MG1655 Δ*rob*-721::*kanR Ptrc10 robH*	*ampR cmR kanR*	*Δrob robH*
1084	*E. coli* MG1655 Δ*yghB*781::*kanR Ptrc*10 *yghB*	*ampR cmR kanR*	*ΔyghB yghB*
1085	*E. coli* MG1655 Δ*yghB*781::*kanR* Ptrc10 empty	*ampR cmR kanR*	*ΔyghB* empty vector
1086	*E. coli* MG1655 Δ*rob*-721::*kanR Ptrc*10 empty	*ampR cmR kanR*	*Δrob* empty vector
1087	*E. coli* MG1655 Δ*marC*750::kanR *Ptrc*10 empty	*ampR cmR kanR*	*ΔmarC* empty vector

Alias is used throughout the manuscript. The reference number refers to the internal strain collection

### Growth/tolerance testing

For plate cultivation, *E. coli* MG1655 was streaked out on M9 medium supplemented with 5 g/L Glucose and grown at 37 °C. When colony formation was observed, 10 mL M9 medium was inoculated with a colony and incubated in a 100 mL baffled flask in a shaking incubator (Multitron or Ecotron, both 25 mm shaking throw at 200 rpm; Infors HT, Bottmingen, Switzerland,). From this culture, an over-night culture of 25 mL medium in a 250 mL baffled flask was inoculated and incubated for 16 h. Inoculation volume was calculated such that the finished culture could be harvested the next morning in the mid-exponential phase. This was used to inoculate 25 mL of medium in 250 mL baffled flasks with Teflon^®^ liner screw cabs to an optical density (OD) of 0.2 and incubated in a shaking incubator as described above. The toxic compound was added to the specified concentration (for isoprenol see below). To characterize the relative fitness of reconstructed strains, growth experiments with butanol (5 g/L) and vanillin (1.5 g/L) were performed separately. OD was measured every hour at 600 nm against water and the growth rate was calculated by a linear fit to log-transformed OD-values. Each experiment was performed in triplicates.

### Evolution

Adaptive evolution was carried out in biological triplicates. 25 mL of M9 medium supplemented with 5 g/l glucose were inoculated with *E. coli* MG1655. Before the cell-culture reached the stationary phase, part of the culture was transferred to fresh medium in a fresh flask with a toxic compound. The mean growth rate of the culture was determined by comparing the initial OD and the culture OD before each passaging. Upon passaging the cell culture, 600 µL was withdrawn and mixed with 600 µL 50% v/v glycerol solution. The samples were stored at – 80 °C.

The initial isoprenol concentration was 60 mM (selected after an initial toxicity screen) and with increasing mean growth rates the isoprenol concentration was increased stepwise to 80 mM isoprenol after approx. 80 generations where it was kept until the end of the experiment.

For easier comparison, the growth rate of evolved or reconstructed strains was divided by the growth rate of the initial MG1655 under the tested conditions and termed ‘relative fitness’.

### Genome re-sequencing

Selected strains were grown overnight in 5 mL lysogeny broth (LB) medium supplemented with 60 mM isoprenol (for mutant strains). Genomic DNA was isolated by LGC Genomics GmbH (Berlin, Germany) using the DNeasy^®^ UltraClean^®^ Microbial Kit (Qiagen, Netherlands). Genomic DNA was fragmented using Covaris (300 bp) (Covaris, MA, USA) and samples were subsequently purified using MinElute columns (Qiagen, Netherlands). Libraries were prepared using Ovation Rapid DR Multiplex System 1–96 (Tecan, Switzerland) and amplified for 13 cycles using MyTaq (Bioline, UK) and standard Illumina primers. Size selection was done on the Pippin Prep system (Sage Science, MA, USA) selecting a range between 300 and 500 bp. Final library purification and quality control of DNA libraries were carried out on BioAnalyzer (Agilent, CA, USA) and Qubit (Thermo Fisher Scientific, MA, USA). Sequencing was done on an Illumina NextSeq 500/550 (Illumina, CA, USA) with 2 × 150 read length following manufacturer’s instructions, achieving about 100× coverage.

Libraries were demultiplexed using Illumina bcl2fastq 2.17.1.14 software [43] followed by clipping of adapters and trimming to achieve a minimum average Phred quality score of 20 over a window of ten bases. Alignment against *E. coli* MG1655 genome was carried out using BWA-MEM version 0.7.12 [44] and variant discovery and genotyping were performed using Freebayes v1.0.2-16 [45].

### RNA-sequencing

The parental MG1655 strain and the three final mutant strains were grown in biological triplicates with 50 mM isoprenol until an OD of 1.0 as described above (25 mL sealed flasks). Then 10 mL of cell-culture was vacuum filtered using a Supor^®^ 800 Grid filter (Pall, NY, USA) with 0.8 µM pore size. The filter containing the cells was put in a 15 mL falcon tube containing 700 µL PGTX solution and immediately frozen in liquid nitrogen. The samples were stored at − 80 °C until further processing.

RNA was extracted using standard methods [46] and RNA quality was determined using Bioanalyzer (Agilent, CA, USA). Ribo-Zero rRNA Removal Kit for Bacteria (Illumina, CA, USA) was used to deplete rRNA. First-strand cDNA synthesis and second-strand synthesis was carried out using NEBNext RNA First Stand and Second Strand Synthesis Module (New England Biolabs, MA, USA). cDNA was then purified and concentrated using MiniElute Columns (Qiagen, Netherlands) and the Encore Rapid DR Multiplex System (Tecan, Switzerland) was used for library preparation. Libraries were amplified for 12 cycles using MyTaq (Bioline, UK) and standard Illumina primers. Size selection was done using a preparative Agarose Gel selecting fragments between 300 and 500 bp and quality control of libraries was performed with Bioanalyzer and Qubit. Finally, sequencing was carried out on Illumina NextSeq500/550 (Illumina, CA, USA) with 1 × 75 bp read length following the manufacturer’s instructions.

The sequencing data were demultiplexed using Illumina bcl2fastq 2.17.1.14 [43], adapters were clipped from the reads, and rRNA was filtered using RiboPicker 0.4.3 [47]. The sequences were aligned using STAR 2.4 and rRNA or tRNA reads were filtered [48]. TopHat-aligned reads were counted using htseq-count [49]. Differential expression was determined using edgeR 3.2.3 [50], DESeq 1.12.0 [51] and cuffdiff 2.1.1 [52]. Raw p-values from the statistical test were adjusted for multiple testing by the Benjamini–Hochberg false discovery rate method [53].

### Expression plasmids and knock-out strains

All plasmids and primer sequences used in this study are provided in the supplementary information (Additional file 1: Tables S8 and S9). Knock-out strains were constructed by amplification of resistance cassette with 25-bp overlap from corresponding Keio strains [42] (Primers 3 + 4, 5 + 6, and 7 + 8). The PCR products carrying a homologous 25-bp sequence and a kanamycin resistance were used to transform *E. coli* MG1655 using standard procedures [42]. For over-expression plasmids, target genes were amplified with a 25-bp homology to the pAH030 overexpression plasmid. The plasmid was linearized using the SpeI restriction site and the PCR-product containing the gene of interest was inserted using Gibson assembly [54]. Recombination was carried out using a standard RED/ET kit [55].

## Supplementary information

**Additional file 1: Table S1.** Mean Growth Rates, Isoprenol Concentration. **Table S2.** Relative Fitness of fast-growing plate colonies.** Figure S1.** Gene expression changes in response to P*yghB* mutation. **Figure S2.** Gene expression changes in the *rob* regulon. **Figure S3.** Clustering of observed genotypes based on Euclidian distance and farthestneighbor (complete linkage clustering).(A) Labeling according to time-point and replicate (B) Labeling with 9 selected mutations. **Table S3.** Genotype labels. **Figure S4.** Proposed model for the evolution of cultures. **Figure S5.** Schematic presentation of implementation of ALE Model. **Figure S6.** Simulation of evolution three adaptive evolution experiments from ALE model. **Figure S7.** Sensitivity analysis of the adapted fitness parameters for the ALE model. **Figure S8.** Sensitivity analysis of the adapted initial proportion parameter Pi for the ALE model. **Table S4.** Likelihood ratio of maximal likelihood estimates of models with contamination compared to reference model without contamination. **Table S5.** Likelihood ratio of maximal likelihood estimates of models with contamination compared to reference model without contamination (different contamination proportions). **Table S6.** GO IDs (by protein) of target mutations. **Table S7.** GO IDs (by protein) of top 20 highly differentially expressed genes in adaptedstrains. **Table S8.** Plasmids. **Table S9.** Primers.

## Data Availability

The evolved strains can be obtained for non-commercial research purposes upon the signature of a materials transfer agreement. Genome re-sequencing data (PRJNA665813) and RNA-sequencing data (GSE158959) has been deposited to the Sequence Read Archive (SRA).

## Abbreviations

ALE: Adaptive laboratory evolution
DXP: 1-Deoxy-d-xylulose 5-phosphate
fs: Frameshift
IPP: Isopentenyl diphosphate
LB: Lysogeny broth
logP: Octanol–water partitioning coefficient
M9: Mineral minimal medium
OD: Optical density
PCR: Polymerase chain reaction
