# Supplementary material for: Evolutionary engineering of E. coli MG1655 for tolerance against isoprenol
Source: Biotechnol Biofuels. 2020 Nov 9;13:183. doi: 10.1186/s13068-020-01825-6 (PMC7653855; doi:10.1186/s13068-020-01825-6)
Supplement: Supplementary file 1 — Additional file 1: Table S1. Mean Growth Rates, Isoprenol Concentration. Table S2. Relative Fitness of fast-growing plate colonies. Figure S1. Gene expression changes in response to PyghB mutation. Figure S2. Gene expression changes in the rob regulon. Figure S3. Clustering of observed genotypes based on Euclidian distance and farthestneighbor (complete linkage clustering).(A) Labeling according to time-point and replicate (B) Labeling with 9 selected mutations. Table S3. Genotype labels. Figure S4. Proposed model for the evolution of cultures. Figure S5. Schematic presentation of implementation of ALE Model. Figure S6. Simulation of evolution three adaptive evolution experiments from ALE model. Figure S7. Sensitivity analysis of the adapted fitness parameters for the ALE model. Figure S8. Sensitivity analysis of the adapted initial proportion parameter Pi for the ALE model. Table S4. Likelihood ratio of maximal likelihood estimates of models with contamination compared to reference model without contamination. Table S5. Likelihood ratio of maximal likelihood estimates of models with contamination compared to reference model without contamination (different contamination proportions). Table S6. GO IDs (by protein) of target mutations. Table S7. GO IDs (by protein) of top 20 highly differentially expressed genes in adaptedstrains. Table S8. Plasmids. Table S9. Primers. [file 13068_2020_1825_MOESM1_ESM.pdf]

Supplementary Information

Evolutionary Engineering of *E. coli* MG1655 for Tolerance Against Isoprenol

Heiko Babel[1,2] and Jens O Krömer[1§]

[1]Systems Biotechnology group, Department of Solar Materials, Helmholtz Centre for Environmental Research - UFZ, Leipzig, Germany

[2]current address: Boehringer Ingelheim Pharma GmbH & Co. KG, Biberach/ Riß, Germany

[§] Corresponding author jens.kroemer@ufz.de

Content:

Table S1: Mean Growth Rates, Isoprenol Concentration 3

Table S2: Relative Fitness of fast-growing plate colonies. 5

Figure S1: Gene expression changes in response to *PyghB* mutation. 6

Figure S2: Gene expression changes in the rob regulon. 8

Supplementary Text: Simple model describes the ALE-experiment........ 10

Figure S3: Clustering of observed genotypes based on Euclidian distance and farthest neighbor (complete linkage clustering). 10

Table S3: Genotype labels 12

Figure S4: Proposed model for the evolution of cultures. 13

Figure S5: Schematic presentation of implementation of ALE Model 15

Figure S6: Simulation of evolution three adaptive evolution experiments from ALE model. 17

Figure S7: Sensitivity analysis of the adapted fitness parameters for the ALE model 18

Figure S8: Sensitivity analysis of the adapted initial proportion parameter Pi for the ALE model 19

Table S4: Likelihood ratio of maximal likelihood estimates of models with contamination compared to reference model without contamination 20

Table S5: Likelihood ratio of maximal likelihood estimates of models with contamination compared to reference model without contamination different contamination proportions. 21

Table S6: GO IDs (by protein) of target mutations 23

Table S7: GO IDs (by protein) of top 20 highly differentially expressed genes in adapted strains..25

Table S8: Plasmids 27

Table S9: Primers 27

Supplementary References..28

Response to Reviews (1st revision)...29

Response to Reviews (2nd revision) 42

Response to Reviews (3rd revision)...52

Table S1: Mean Growth Rates, Isoprenol Concentration

Optical density was determined at the beginning at the end of each passage and growth rate was calculated from these two values and the parallel cultures averaged. This provides a qualitative estimate of growth since cells might have already reached stationary phase. It was considered the lower limit for the true exponential growth rate. 'Restart' indicates the cryo stocks that were used to restart the evolution after extinction by 90 mM isoprenol.

Sequencing	Date	Generations	Mean Growth rate [1/h]	Isoprenol [mM]
	22.03.2018	4	0.117	60
	23.03.2018	5	0.027	60
	27.03.2018	13	0.142	60
	28.03.2018	19	0.174	60
	29.03.2018	26	0.193	60
T1	30.03.2018	32	0.186	64
	02.04.2018	39	0.068	66
	03.04.2018	43	0.101	66
	04.04.2018	49	0.189	65.83

	05.04.2018	56	0.187	68
T2	06.04.2018	62	0.177	72
	09.04.2018	69	0.068	74
	11.04.2018	79	0.172	76
	12.04.2018	84	0.143	80
	13.04.2018	89	0.161	80
	17.04.2018	99	0.067	80
	18.04.2018	103	0.119	80
T3	19.04.2018	108	0.170	80
	20.04.2018	114	0.152	80
	23.04.2018	121	0.062	80
	24.04.2018	122	0.032	80
T4	25.04.2018	126	0.130	80
	26.04.2018	132	0.162	80
	27.04.2018	137	0.173	80
Restart	30.04.2018	149	0.102	80
T5	21.05.2018	149	n.d.	80
	24.05.2018	158	0.067	80
	27.05.2018	164	0.058	80
	30.05.2018	169	0.070	80
	31.05.2018	173	0.095	80
T6	01.06.2018	177	0.140	80
	02.06.2018	183	0.157	80
	03.06.2018	188	0.154	80
	04.06.2018	193	0.132	80
	05.06.2018	198	0.145	80
	06.06.2018	203	0.178	80
	07.06.2018	207	0.113	80
	15.06.2018	220	0.033	80
T7	18.06.2018	226	0.066	80

n.d.= not determined

Table S2: Relative Fitness of fast-growing plate colonies.

Cells were incubated overnight in liquid culture (falcon tube assay) at 60 mM Isoprenol.

Clones used for sequencing are indicated in bold.

Generation	32	62	108	126	149	177	226
A-1	1.21	1.08	0.96	1.05	1.50	1.41	1.22
A-2	1.17	0.98	0.99	0.94	**1.59**	**1.60**	1.18
A-3	1.14	1.15	1.06	1.13	1.51	1.28	**1.34**
A-4	1.22	**1.19**	**1.10**	1.02	1.37	1.53	1.19
A-5	**1.30**	1.05	1.09	**1.14**	1.59	1.60	n.d.
B-1	1.18	1.19	**1.13**	1.15	1.35	1.41	1.22
B-2	1.15	**1.28**	1.05	1.25	1.15	**1.59**	**1.34**
B-3	1.18	1.00	0.98	1.24	**1.36**	1.43	1.22
B-4	**1.32**	1.10	1.02	1.11	1.29	1.57	1.17
B-5	1.26	1.05	1.00	**1.30**	1.26	1.37	1.25
C-1	1.23	1.15	1.10	1.08	1.45	1.30	1.15
C-2	1.16	0.96	1.11	0.98	**1.69**	1.39	1.31
C-3	**1.25**	1.04	1.13	1.07	1.54	**1.47**	1.16
C-4	1.18	**1.24**	**1.16**	0.98	1.47	1.26	**1.33**
C-5	1.16	1.08	1.08	**1.22**	1.47	1.41	1.22

n.d.= not determined

Figure S1: Gene expression changes in response to *PyghB* mutation.

A

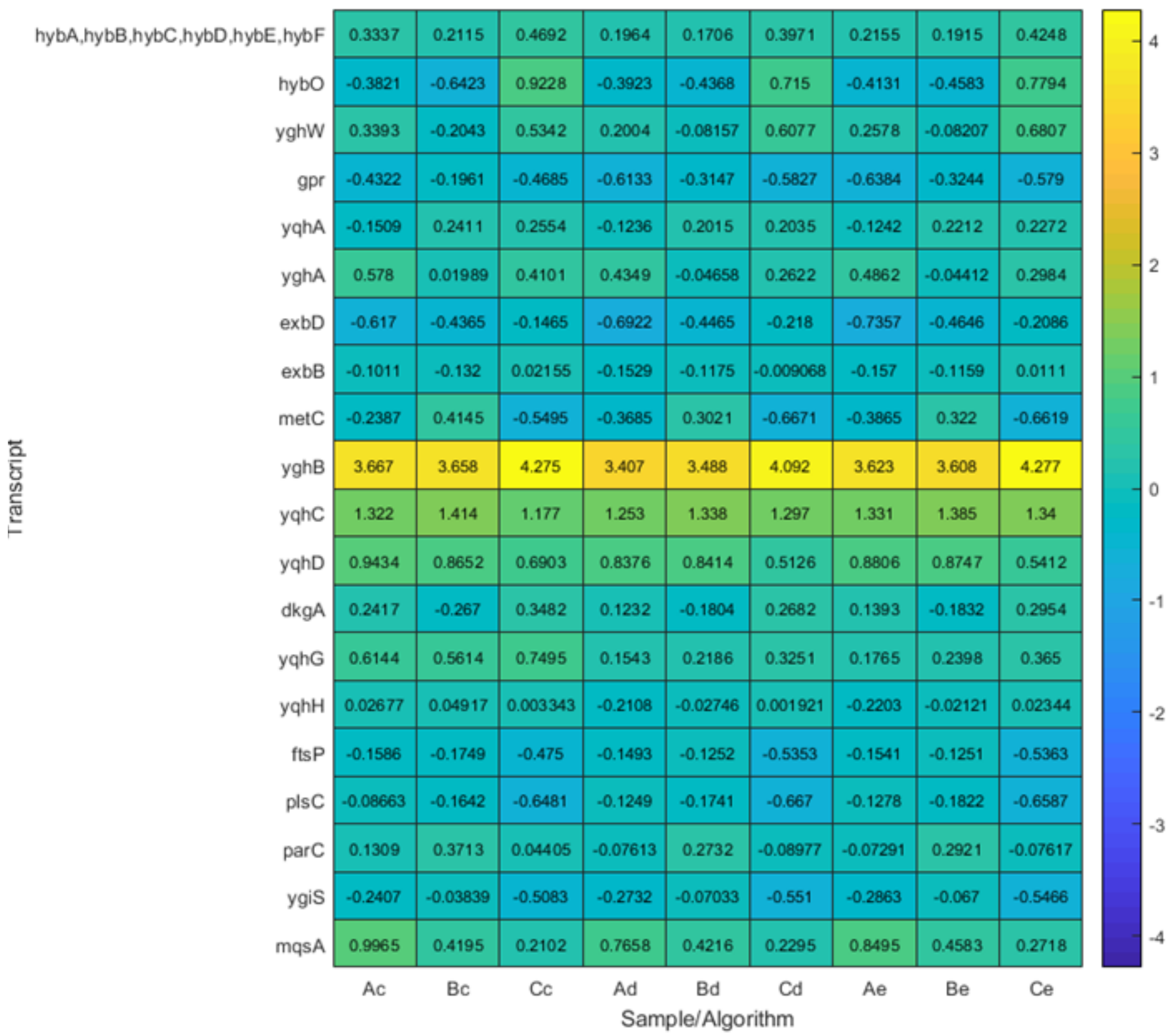

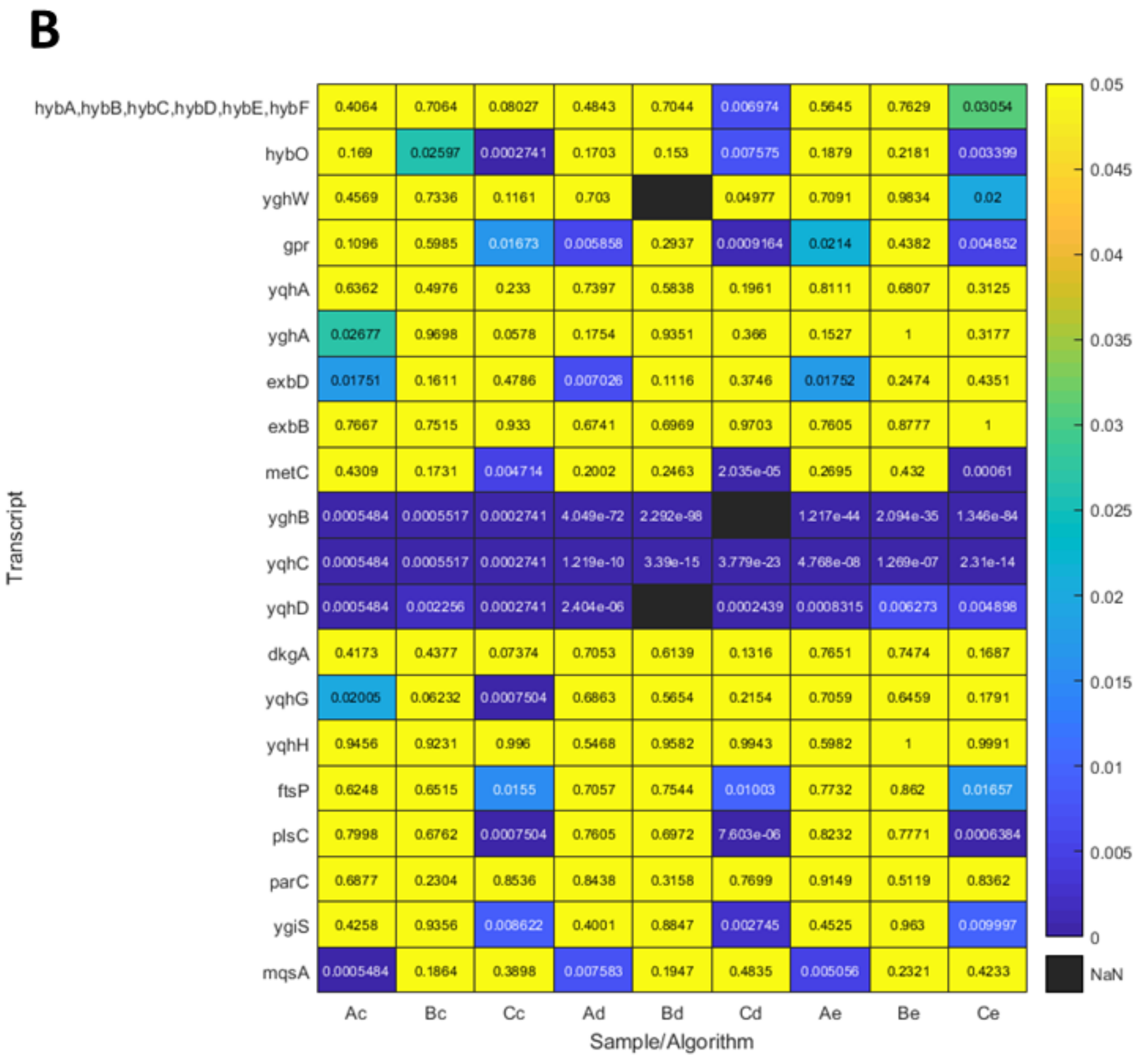

Fig S1: Changes in gene expression in the downstream and upstream region of the mutation in the promotor region of *yghB*. *(A) Fold change for different DE algorithms (B) Significance tests of fold-change (p-values color-coded to scale on the right).*

Figure S2: Gene expression changes in the rob regulon.

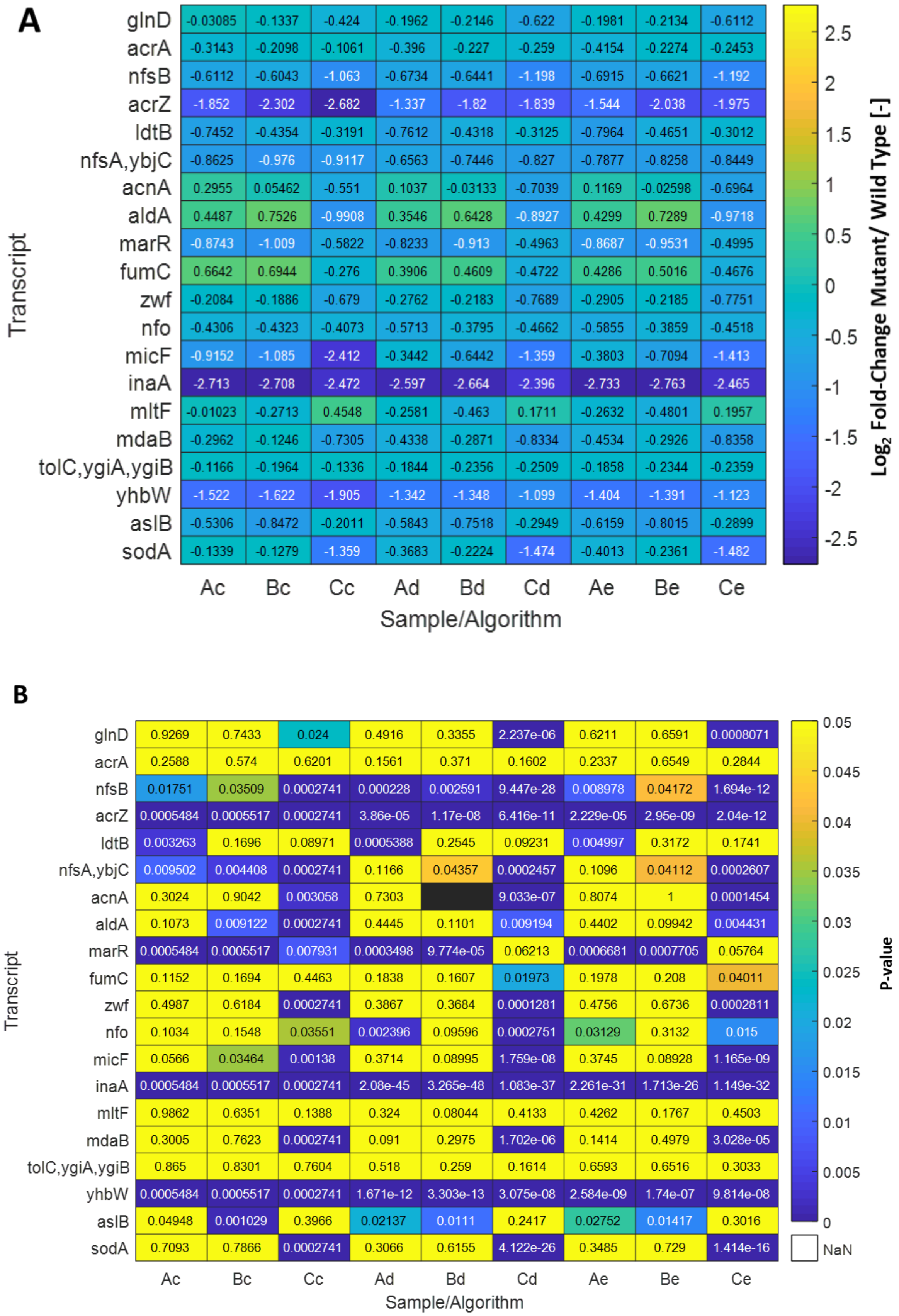

Fig S2: Changes in gene expression in rob regulon. *(A) Fold change for different DE algorithms (B) Significance tests of fold-change.*

Supplementary Text: Simple model describes the ALE-experiment

Since we observed very similar genotypes after T5 we wondered if this could be expected of an adaptive laboratory evolution experiment. As a first step, we analyzed the observed genotype landscape by hierarchical clustering. Indeed, the observed genotypes can be clustered into two major clusters, a cluster close to the wild-type and a cluster containing the final genotypes (supplementary figure S3A).

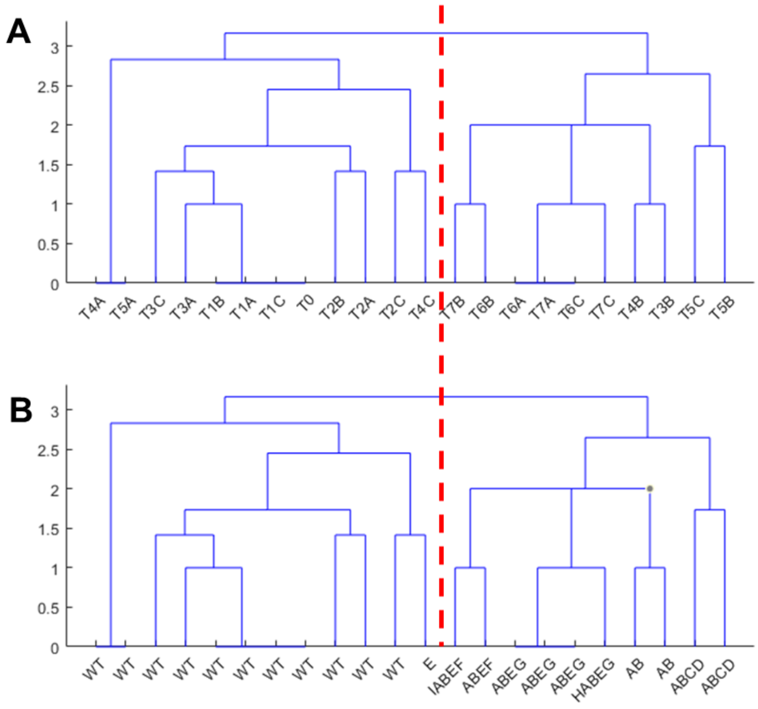

Figure S3: Clustering of observed genotypes based on Euclidian distance and farthest neighbor (complete linkage clustering).

(A) Labeling according to time-point and replicate (B) Labeling with 9 selected mutations

To reconstruct the occurrence of the final genotypes, we only focus on the mutations present in the final genotypes; intermittent genotypes are assumed to not have a large fitness benefit and therefore can be considered similar to the wild type. After labeling

the dendrogram with the final mutations (according to the labeling in supplementary table S7), we can construct a hypothesis for the occurrence of the final genotypes.

Table S3: Genotype labels

Mutation	Label
fabF 1	A
marC 3	B
iscR	C
trkH	D
P_{yghB}	E
rob 2	F
rob 3	G
frmR 1	H
frmR 2	I

If we root the dendrogram in supplementary figure S3B in the wild-type WT we can derive a hypothetical occurrence of genotypes presented in supplementary figure S4 Here the wild-type WT can mutate to genotype A or E. A consequently mutates to AB and can mutate to ABC and ABE. ABC then mutates to ABCD. ABE can mutate to final genotypes ABEG and ABEF which can further mutate to ABEGH and ABEFI.

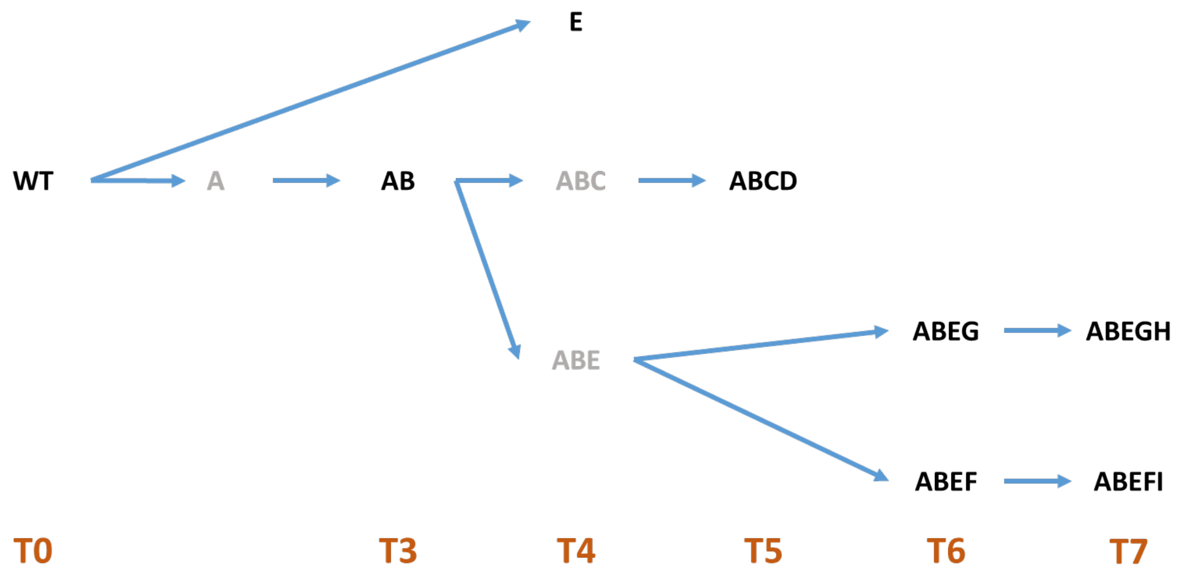

Figure S4: Proposed model for the evolution of cultures.

Black labels indicate observed genotypes, gray labels are proposed intermediate genotypes.

With this hypothesis, we implemented a simple model in MATLAB R2019a based on a more comprehensive model by LaCroix et. al. [2]. The model simulates proportions of genotypes in cultures during an experimental evolution experiment. It is based on the following assumptions:

1. Similar beneficial mutations occur during an ALE experiment [2–4]
    - This is reasonable since we observe exactly the same mutations that have been described in the literature previously (fabF).
2. Evolutionary bottleneck during passaging can be neglected [2]
3. Beneficial mutations occur per cultivation.
    - This is reasonable given literature values of beneficial mutations rates. [2]
4. Number of cumulated generations per cultivation is constant.

    - Given the increased stress level during our actual experiment, this is also a reasonable assumption.
5. No back-mutation
    - This means that all mutated strains need to outcompete each other to prevail.
6. Genotypes not listed in supplementary table 1 do not have a significant fitness advantage.

The implementation of this model is depicted in supplementary figure S5.

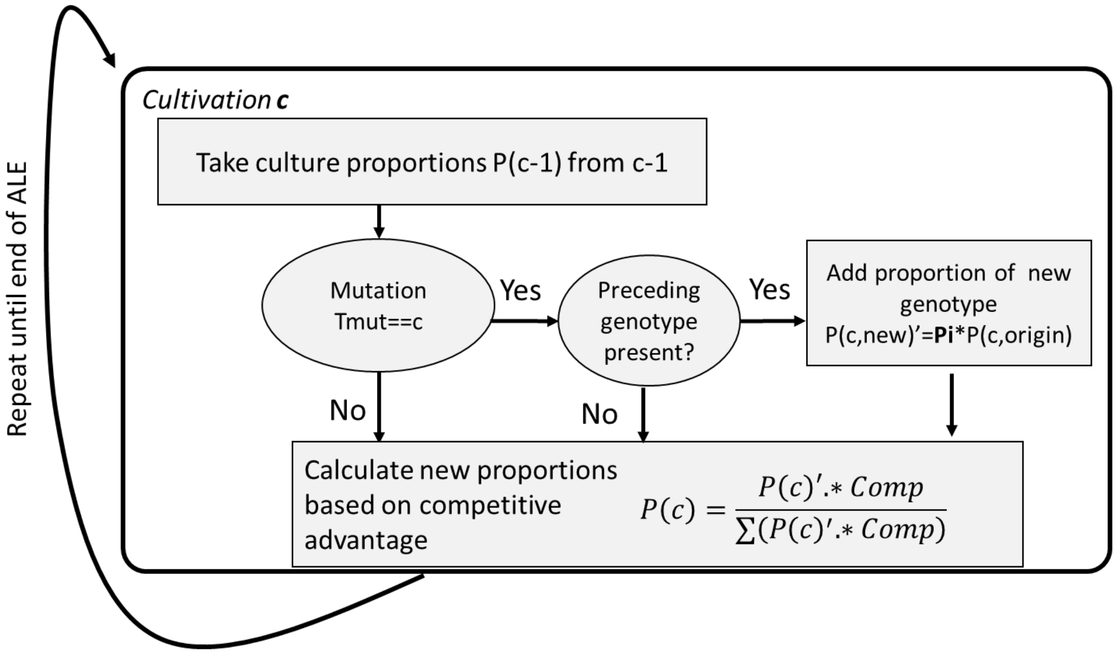

Proportions Matrix **P**

Cultivation	WT	A	AB	E	...
1	1	0	0	0	
2	0.9	0.1	0	0	
3	0.8	0.15	0	0.05	
...					

Competitive Advantage Vector **Comp**

WT	A	AB	...
1	$e^{nfAln(2)}$	$e^{nfABln(2)}$	...

Mutation Vector **Mut**

Tmut	Origin Genotype	Mutated Genotype
m	WT	A
n	A	AB
o	WT	E
...		

Figure S5: Schematic presentation of implementation of ALE Model

The model should explain all observations, i.e. all isolated genotypes in all biological replicate evolutions. To this end three parameter sets need to be adapted, the competitive fitness advantage **Comp** of the genotypes, the time-point of occurrence of a mutation in a culture **Tmut** and the initial proportion of the new genotype **Pi**. The initial proportion **Pi** correlates with the beneficial mutation rate. The competitive advantage **Comp** is derived from the proportional increase of a mutant genotype with increased fitness $\mu = \mu_{WT}\ (1 + f)$ compared to wild-type (during exponential growth):

$$Comp = \frac{e^{t*\mu_{WT}*(1+f)}}{e^{t*\mu_{WT}}} = e^{t*\mu_{WT}*f} = e^{n_{gen\ in\ cult}*\ln(2)*f}$$

The initial proportion **Pi** and the competitive fitness of a genotype **Comp** are global parameters and are valid for all three replicates.

We assume that the likelihood L of isolating a genotype is proportional to the proportion of a genotype at that time-point in the experiment in the culture:

$$L(isolated\ genotype) \propto propotion(observed\ genotype\ in\ culture)$$

This would be the case if cultures were isolated on a medium without selected pressure at random. This gives us a lower limit of the true likelihood since in the experimental conditions there was an additional selection step during the isolation. The true likelihood of the observation is therefore higher. Derived from the likelihood of observing an isolated genotype we can directly derive the maximum likelihood estimator (MLE):

$$MLE \propto argmax_P \prod_{i}^{N_{Replicate}} \prod_{j}^{M_{time-point}} propotion(observed\ genotpye_{i,j})$$

Maximum-likelihood estimates were obtained by using the MATLAB function *fminsearch* (minimizing the negative log-likelihood) with 100 different random initial parameters. The results of the simulation are shown in supplementary figure S6.

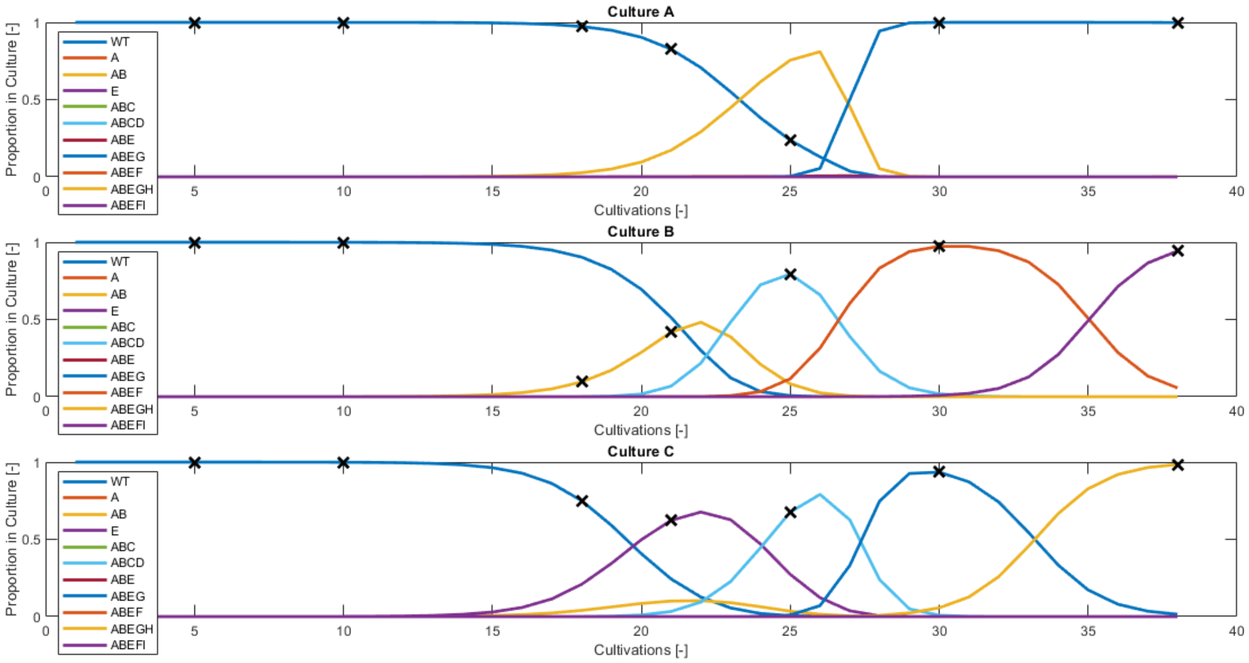

Figure S6: Simulation of evolution three adaptive evolution experiments from ALE model. ***X*** *indicates proportion of observed genotype. The mean number of generations in each cultivation is* $n_{gen\ in\ cult} = 6$.

The proposed model can describe the occurrence of the isolated genotypes. In this simulation the isolated genotypes are the genotypes with the highest proportion. At two time-points in culture B the wild-type has a higher proportion then the isolated genotype AB, however, this can readily be explained by the additional selection step in the isolation procedure. In those cases, the genotype AB outcompetes the wild-type during the selection procedure. The good fit of the model to the experimental data shows that no other mechanism then adaptive evolution is necessary to fully explain the experimental results.

Due to the non-linear high dimensional nature of the problem, the model might fit the data well without having well-defined parameters (non-identifiability). To exclude this effect, we conducted a sensitivity analysis where we randomly varied the starting

values of the adaptation algorithm with 20 % normally distributed standard deviation. As shown in supplementary figure S7 and S8 the algorithm always yields narrow distributions of the adapted parameter values. The adapted parameter values do not exhibit any significant tailing. Also, we find that more complex genotypes (that correspond to the final phenotypes) have a higher competitive fitness value (supplementary figure S7).

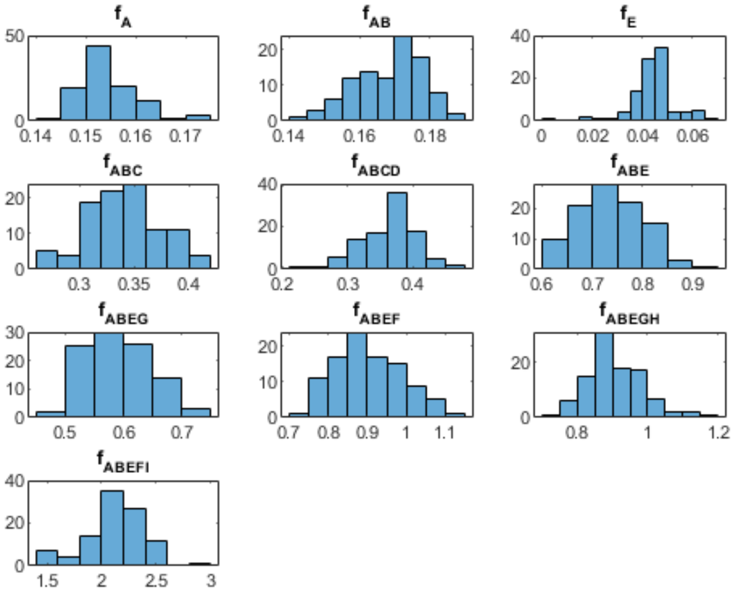

Figure S7: Sensitivity analysis of the adapted fitness parameters for the ALE model.

Histogram of optimal parameters identified by 100 parameter adaptation runs from random initial conditions.

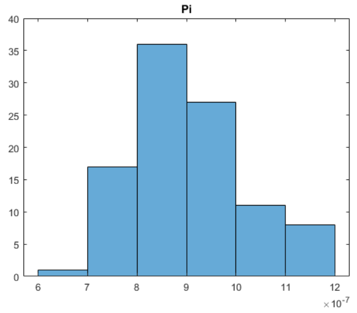

Figure S8: Sensitivity analysis of the adapted initial proportion parameter Pi for the ALE model. *Histogram of optimal parameter identified by 100 parameter adaptation runs from random initial conditions.*

This identifiable and descriptive model was the basis for further analysis of the evolution data. We wanted to systematically investigate the effect of adding the occurrence of a contamination event. To this end, the model was executed until the time-point after which the contamination event should occur. To simulate the contamination the mutant composition of the contaminating culture was added to the mutant composition of the contaminated cultures. Finally, the maximum likelihood estimate for each model was determined. This value was used to calculate a likelihood ratio given by $LR = \frac{MaximumLikelihood(No\ Contamination)}{MaximumLikelihood(Contamination)}$.

The results of this analysis for a scenario of 5% contamination are shown in Table S8 (additional values are given in Table S9). For some contamination models, there is indeed a likelihood ratio below 1, indicating a higher likelihood of a model with contamination compared to a model without contamination. The lowest likelihood-ratio is observed for a scenario in which Culture C contaminates Culture A and B after T5. However, the results of a likelihood ratio test with a Chi-squared distribution (Wilk's Theorem) show that none of the contamination models are significantly different from

the model without contamination. We conclude that, on the basis of the given data no distinction between the model with or without contamination can be made.

Table S4: Likelihood ratio of maximal likelihood estimates of models with contamination compared to reference model without contamination. *Lower values indicate higher likelihood of contamination model. P-values are calculated based on a Chi^2 distribution with n-p=10 degrees of freedom and given in brackets. Time-points indicate the sampling time-point after which the contamination occurs. Culture refers to the culture from which the contamination originates. Proportion of contamination 0.05.*

	Culture A	Culture B	Culture C
T1	1.046 (1.00)	0.286 (0.99)	0.343 (1.00)
T2	1.050 (1.00)	0.284 (0.99)	2.119 (1.00)
T3	1.020 (1.00)	0.243 (0.99)	0.442 (1.00)
T4	0.988 (1.00)	0.199 (0.98)	0.077 (0.88)
T5	1.001 (1.00)	1.004 (1.00)	0.040 (0.78)
T6	1.006 (1.00)	0.986 (1.00)	0.960 (1.00)

Table S5: Likelihood ratio of maximal likelihood estimates of models with contamination compared to reference model without contamination different contamination proportions. *Lower values indicate higher likelihood of contamination model. P-values are calculated based on a Chi^2 distribution with n-p=10 degrees of freedom and given in brackets. Time-points indicate the sampling time-point after which the contamination occurs. Culture refers to the culture from which the contamination originates.*

	Culture A	Culture B	Culture C
Proportion of Contamination = 0.10			
T1	1.109 (1.00)	0.463 (1.00)	0.666 (1.00)
T2	1.070 (1.00)	0.465 (1.00)	0.993 (1.00)
T3	1.061 (1.00)	0.360 (1.00)	0.736 (1.00)
T4	1.005 (1.00)	0.126 (0.94)	0.422 (1.00)
T5	0.999 (1.00)	1.016 (1.00)	0.036 (0.76)
T6	1.052 (1.00)	0.502 (1.00)	0.952 (1.00)
Proportion of Contamination = 0.25			
T1	1.295 (1.00)	1.043 (1.00)	2.521 (1.00)
T2	1.289 (1.00)	1.038 (1.00)	1.269 (1.00)
T3	1.184 (1.00)	2.042 (1.00)	1.932 (1.00)
T4	1.012 (1.00)	1.117 (1.00)	1.106 (1.00)
T5	1.013 (1.00)	1.034 (1.00)	0.036 (0.76)
T6	0.950 (1.00)	0.498 (1.00)	1.012 (1.00)
Proportion of Contamination = 0.50			
T1	1.807 (1.00)	2.121 (1.00)	2.917 (1.00)
T2	1.828 (1.00)	2.650 (1.00)	7.887 (1.00)

T3	1.601 (1.00)	2.385 (1.00)	6.399 (1.00)
T4	1.029 (1.00)	2.137 (1.00)	2.910 (1.00)
T5	1.020 (1.00)	0.990 (1.00)	0.036 (0.76)
T6	1.023 (1.00)	0.989 (1.00)	0.994 (1.00)
Proportion of Contamination = 0.75			
T1	2.861 (1.00)	4.273 (1.00)	16.315 (1.00)
T2	2.966 (1.00)	4.170 (1.00)	15.805 (1.00)
T3	2.393 (1.00)	5.567 (1.00)	12.215 (1.00)
T4	1.124 (1.00)	4.594 (1.00)	1.630 (1.00)
T5	1.046 (1.00)	1.032 (1.00)	0.041 (0.78)
T6	0.544 (1.00)	0.994 (1.00)	1.065 (1.00)
Proportion of Contamination = 0.90			
T1	4.727 (1.00)	5.491 (1.00)	22.595 (1.00)
T2	4.114 (1.00)	5.471 (1.00)	22.218 (1.00)
T3	5.378 (1.00)	14.965 (1.00)	13.616 (1.00)
T4	1.255 (1.00)	1.484 (1.00)	11.785 (1.00)
T5	1.001 (1.00)	1.097 (1.00)	0.056 (0.83)
T6	1.061 (1.00)	0.981 (1.00)	0.614 (1.00)
Proportion of Contamination = 0.95			
T1	4.589 (1.00)	5.641 (1.00)	24.306 (1.00)
T2	5.222 (1.00)	6.014 (1.00)	26.968 (1.00)
T3	11.049 (1.00)	27.247 (1.00)	13.557 (1.00)
T4	1.456 (1.00)	9.163 (1.00)	15.026 (1.00)
T5	0.987 (1.00)	1.351 (1.00)	0.041 (0.78)
T6	1.098 (1.00)	1.023 (1.00)	1.098 (1.00)

Table S6: GO IDs (by protein) of target mutations

Code	Name	Percentage	Count
GO:0005886	plasma membrane	41	7
GO:0005829	cytosol	41	7
GO:0016020	membrane	41	7
GO:0005515	protein binding	35	6
GO:0016021	integral component of membrane	35	6
GO:0005737	cytoplasm	29	5
GO:0005887	integral component of plasma membrane	29	5
GO:0016740	transferase activity	29	5
GO:0006355	regulation of transcription, DNA-templated	24	4
GO:0003677	DNA binding	24	4
GO:0003700	DNA-binding transcription factor activity	18	3
GO:0003824	catalytic activity	18	3
GO:0006629	lipid metabolic process	18	3
GO:0046872	metal ion binding	18	3
GO:0008654	phospholipid biosynthetic process	12	2
GO:0006633	fatty acid biosynthetic process	12	2
GO:0005524	ATP binding	12	2
GO:0042802	identical protein binding	12	2
GO:0016746	transferase activity, transferring acyl groups	12	2
GO:0043565	sequence-specific DNA binding	12	2
GO:0000166	nucleotide binding	12	2
GO:0006631	fatty acid metabolic process	12	2
GO:0016747	transferase activity, transferring acyl groups other than amino-acyl groups	12	2
GO:0045892	negative regulation of transcription, DNA-templated	12	2
GO:0055085	transmembrane transport	12	2
GO:0004004	ATP-dependent RNA helicase activity	6	1
GO:0004366	glycerol-3-phosphate O-acyltransferase activity	6	1
GO:0000027	ribosomal large subunit assembly	6	1
GO:0006099	tricarboxylic acid cycle	6	1
GO:0009401	phosphoenolpyruvate-dependent sugar phosphotransferase system	6	1
GO:0033817	beta-ketoacyl-acyl-carrier-protein synthase II activity	6	1
GO:0000155	phosphorelay sensor kinase activity	6	1
GO:0003690	double-stranded DNA binding	6	1
GO:0006644	phospholipid metabolic process	6	1
GO:0008428	ribonuclease inhibitor activity	6	1
GO:0015796	galactitol transport	6	1
GO:0016310	phosphorylation	6	1
GO:0023014	signal transduction by protein phosphorylation	6	1
GO:0071805	potassium ion transmembrane transport	6	1
GO:0000160	phosphorelay signal transduction system	6	1
GO:0000986	bacterial-type proximal promoter sequence-specific DNA binding	6	1
GO:0003676	nucleic acid binding	6	1
GO:0003723	RNA binding	6	1
GO:0004108	citrate (Si)-synthase activity	6	1
GO:0004315	3-oxoacyl-[acyl-carrier-protein] synthase activity	6	1
GO:0004386	helicase activity	6	1
GO:0004673	protein histidine kinase activity	6	1
GO:0005267	potassium channel activity	6	1
GO:0005506	iron ion binding	6	1

GO:0006071	glycerol metabolic process	6	1
GO:0006811	ion transport	6	1
GO:0006812	cation transport	6	1
GO:0006813	potassium ion transport	6	1
GO:0007165	signal transduction	6	1
GO:0007584	response to nutrient	6	1
GO:0008143	poly(A) binding	6	1
GO:0008152	metabolic process	6	1
GO:0008186	RNA-dependent ATPase activity	6	1
GO:0008324	cation transmembrane transporter activity	6	1
GO:0008374	O-acyltransferase activity	6	1
GO:0008643	carbohydrate transport	6	1
GO:0015379	potassium:chloride symporter activity	6	1
GO:0015577	galactitol transmembrane transporter activity	6	1
GO:0016024	CDP-diacylglycerol biosynthetic process	6	1
GO:0016301	kinase activity	6	1
GO:0016772	transferase activity, transferring phosphorus-containing groups	6	1
GO:0016787	hydrolase activity	6	1
GO:0016887	ATPase activity	6	1
GO:0018106	peptidyl-histidine phosphorylation	6	1
GO:0019402	galactitol metabolic process	6	1
GO:0019899	enzyme binding	6	1
GO:0022857	transmembrane transporter activity	6	1
GO:0030955	potassium ion binding	6	1
GO:0033592	RNA strand annealing activity	6	1
GO:0034219	carbohydrate transmembrane transport	6	1
GO:0042254	ribosome biogenesis	6	1
GO:0043093	FtsZ-dependent cytokinesis	6	1
GO:0044255	cellular lipid metabolic process	6	1
GO:0046912	transferase activity, transferring acyl groups, acyl groups converted into alkyl on transfer	6	1
GO:0051252	regulation of RNA metabolic process	6	1
GO:0051536	iron-sulfur cluster binding	6	1
GO:0051537	2 iron, 2 sulfur cluster binding	6	1
GO:0060698	endoribonuclease inhibitor activity	6	1
GO:0060699	regulation of endoribonuclease activity	6	1
GO:0060702	negative regulation of endoribonuclease activity	6	1
GO:0090584	protein-phosphocysteine-galactitol-phosphotransferase system transporter activity	6	1
GO:0102420	sn-1-glycerol-3-phosphate C16:0-DCA-CoA acyl transferase activity	6	1
GO:1902369	negative regulation of RNA catabolic process	6	1

Table S7: GO IDs (by protein) of top 20 highly differentially expressed genes in adapted strains

Code	Name	Percentage	Count
GO:0016020	membrane	61.54	8
GO:0016021	integral component of membrane	53.85	7
GO:0005886	plasma membrane	38.46	5
GO:0005515	protein binding	30.77	4
GO:0005887	integral component of plasma membrane	30.77	4
GO:0005524	ATP binding	23.08	3
GO:0000166	nucleotide binding	23.08	3
GO:0000287	magnesium ion binding	23.08	3
GO:0016740	transferase activity	23.08	3
GO:0009279	cell outer membrane	15.38	2
GO:0042802	identical protein binding	15.38	2
GO:0008652	cellular amino acid biosynthetic process	15.38	2
GO:0009082	branched-chain amino acid biosynthetic process	15.38	2
GO:0009097	isoleucine biosynthetic process	15.38	2
GO:0009099	valine biosynthetic process	15.38	2
GO:0009234	menaquinone biosynthetic process	7.69	1
GO:0008771	[citrate (pro-3S)-lyase] ligase activity	7.69	1
GO:0015288	porin activity	7.69	1
GO:0034220	ion transmembrane transport	7.69	1
GO:0005737	cytoplasm	7.69	1
GO:0009376	HslUV protease complex	7.69	1
GO:0016887	ATPase activity	7.69	1
GO:0034639	L-amino acid efflux transmembrane transporter activity	7.69	1
GO:0046428	1,4-dihydroxy-2-naphthoate octaprenyltransferase activity	7.69	1
GO:1902475	L-alpha-amino acid transmembrane transport	7.69	1
GO:0003984	acetolactate synthase activity	7.69	1
GO:0004659	prenyltransferase activity	7.69	1
GO:0005216	ion channel activity	7.69	1
GO:0005576	extracellular region	7.69	1
GO:0005829	cytosol	7.69	1
GO:0006464	cellular protein modification process	7.69	1
GO:0008887	glycerate kinase activity	7.69	1
GO:0042597	periplasmic space	7.69	1
GO:0043335	protein unfolding	7.69	1
GO:0046296	glycolate catabolic process	7.69	1
GO:0046930	pore complex	7.69	1
GO:0070207	protein homotrimerization	7.69	1
GO:1902201	negative regulation of bacterial-type flagellum-dependent cell motility	7.69	1
GO:0001530	lipopolysaccharide binding	7.69	1
GO:0003824	catalytic activity	7.69	1
GO:0004176	ATP-dependent peptidase activity	7.69	1
GO:0006508	proteolysis	7.69	1
GO:0006811	ion transport	7.69	1
GO:0006855	drug transmembrane transport	7.69	1
GO:0006865	amino acid transport	7.69	1
GO:0008080	N-acetyltransferase activity	7.69	1
GO:0008219	cell death	7.69	1

GO:0008289	lipid binding	7.69	1
GO:0009058	biosynthetic process	7.69	1
GO:0009408	response to heat	7.69	1
GO:0009436	glyoxylate catabolic process	7.69	1
GO:0009986	cell surface	7.69	1
GO:0010676	positive regulation of cellular carbohydrate metabolic process	7.69	1
GO:0015075	ion transmembrane transporter activity	7.69	1
GO:0015299	solute:proton antiporter activity	7.69	1
GO:0015489	putrescine transmembrane transporter activity	7.69	1
GO:0015808	L-alanine transport	7.69	1
GO:0015847	putrescine transport	7.69	1
GO:0016301	kinase activity	7.69	1
GO:0016310	phosphorylation	7.69	1
GO:0016765	transferase activity, transferring alkyl or aryl (other than methyl) groups	7.69	1
GO:0016874	ligase activity	7.69	1
GO:0019904	protein domain specific binding	7.69	1
GO:0030976	thiamine pyrophosphate binding	7.69	1
GO:0031388	organic acid phosphorylation	7.69	1
GO:0032194	ubiquinone biosynthetic process via 3,4-dihydroxy-5-polyprenylbenzoate	7.69	1
GO:0032973	amino acid export across plasma membrane	7.69	1
GO:0034214	protein hexamerization	7.69	1
GO:0034702	ion channel complex	7.69	1
GO:0035442	dipeptide transmembrane transport	7.69	1
GO:0042371	vitamin K biosynthetic process	7.69	1
GO:0042908	xenobiotic transport	7.69	1
GO:0042910	xenobiotic transmembrane transporter activity	7.69	1
GO:0042912	colicin transmembrane transporter activity	7.69	1
GO:0043190	ATP-binding cassette (ABC) transporter complex	7.69	1
GO:0043213	bacteriocin transport	7.69	1
GO:0043798	glycerate 2-kinase activity	7.69	1
GO:0047485	protein N-terminus binding	7.69	1
GO:0055085	transmembrane transport	7.69	1
GO:0070011	peptidase activity, acting on L-amino acid peptides	7.69	1
GO:0071916	dipeptide transmembrane transporter activity	7.69	1
GO:0097718	disordered domain specific binding	7.69	1
GO:1900191	negative regulation of single-species biofilm formation	7.69	1
GO:1900232	negative regulation of single-species biofilm formation on inanimate substrate	7.69	1
GO:1902600	proton transmembrane transport	7.69	1

Table S8: Plasmids

Plasmid	Genotype	Reference
pAH030 (SOMA81)	P_{trc10} *ori(pUC) cm*^{R} *amp*^{R} *lacI*	[1]
pHB01	P_{trc10} *yghB ori(pUC) cm*^{R} *amp*^{R} *lacI*	This study
pHB02	P_{trc10} *rob*H48-fs *ori(pUC) cm*^{R} *amp*^{R} *lacI*	This study
pHB03	P_{trc10} *marC* M35-stop *ori(pUC) cm*^{R} *amp*^{R} *lacI*	This study

Table S9: Primers

Primer No	Name	Sequence
1	yghB expr fwd	GGATAACAATTTCACACATACTAGT CGCTGTTCCACAGGAAAGTCC
2	yghB expr rev	CTTTCGTTTTATTTGATGCCTGGTACCTAGGCCGGGAACGGGGAAAATCG
3	rob del fwd	AATTACCTGATGTCAGGTGCTCGTTGTTGAAAGGATGAGGATATTTTATG
4	rob del rev	GACGCCCCTGCATTAGATGAGCTGCAGCGTTAACGACGGATCGGAATCAG
5	marC del fwd	CTTATACTTTTCGCTGATAACCCAGATACACAGGATAACAACCACCAATG
6	marC del rev	AATAGTTGAAAGGCCCATTCGGGCCTTTTTTAATGGTACGTTTTAATGAT
7	yghB del fwd	GTACAATAGGCAGATAAAGGCTTAAACGCTGTTCCACAGGAAAGTCCATG
8	yghB del rev	CGGTACAGCAACCGGGAACGGGAAAATCGTCAGGCGTTACAGTATTTTTT
13	rob H48 fwd	GGATAACAATTTCACACATACTAGTCCTGATGTCAGGTGCTCGTT
14	rob H48 rev	CTTTCGTTTTATTTGATGCCTGGTACCTAGGTCACGAATACCAAAGGCGCTCCA
15	marC M35 fwd	GGATAACAATTTCACACATACTAGTTACACAGGATAACAACCACCAATG
16	marC M35 rev	CTTTCGTTTTATTTGATGCCTGGTACCTAGGTCAGTTGCCTGCCAGGCCAA

Supplementary References

1. Hoschek A, Bühler B, Schmid A. Overcoming the Gas-Liquid Mass Transfer of Oxygen by Coupling Photosynthetic Water Oxidation with Biocatalytic Oxyfunctionalization. Angew. Chemie Int. Ed. 2017;56:15146–9.

2. LaCroix RA, Palsson BO, Feist AM. A Model for Designing Adaptive Laboratory Evolution Experiments. Kivisaar M, editor. Appl. Environ. Microbiol. 2017;83:e03115-16.

3. Perfeito L, Fernandes L, Mota C, Gordo I. Adaptive mutations in bacteria: High rate and small effects. Science (80-.). American Association for the Advancement of Science; 2007;317:813–5.

4. LaCroix RA, Sandberg TE, O'Brien EJ, Utrilla J, Ebrahim A, Guzman GI, et al. Use of adaptive laboratory evolution to discover key mutations enabling rapid growth of Escherichia coli K-12 MG1655 on glucose minimal medium. Appl. Environ. Microbiol. American Society for Microbiology; 2015;81:17–30.
